# Supplementary material for: Pan-cancer characterisation of microRNA across cancer hallmarks reveals microRNA-mediated downregulation of tumour suppressors
Source: Nat Commun. 2018 Dec 7;9:5228. doi: 10.1038/s41467-018-07657-1 (PMC6286392; doi:10.1038/s41467-018-07657-1)
Supplement: Supplementary file 1 — Supplementary Information [file 41467_2018_7657_MOESM1_ESM.pdf]

Supplementary Information and Supplementary Figure Legends

Pan-cancer characterisation of microRNA with hallmarks of cancer reveals role of microRNA-mediated downregulation of tumour suppressor genes

Andrew Dhawan[1], Jacob G. Scott[2], Adrian L. Harris[1], and Francesca M. Buffa[1*]

1 Department of Oncology, University of Oxford, Oxford, United Kingdom
2 Translational Hematology and Radiology, Cleveland Clinic, Cleveland, United States

*** francesca.buffa@oncology.ox.ac.uk**

Supplementary Note 1. Listing of genes included in each gene signature, COSMIC tumour suppressor genes, and oncogenes, supplementary methods figure

The listing for the Entrez IDs used for all gene signatures considered are provided in the text files contained within the gene_signatures subfolder of the supplementary .zip file. The lists of COSMIC tumour suppressor genes and oncogenes may be found in text files within the COSMIC subfolder within the supplementary .zip file.
We also include here a flow diagram of the methodological approach taken in the main text for identifying TSG-miRNA pairs showing significant repression across cancer types, in association with the hallmark gene signatures.

Supplementary Note 2. sigQC Gene signature quality control summary plots

Here, we present radar plots summarising the various gene signature quality control metrics implemented by the sigQC R package, developed by Dhawan et al. [1]. In general, signature quality is reflected by the overall closeness to the outer rim of the radar plot, for each of the 14 metrics considered. Supplementary figures 2-10 contain signature quality control plots grouped by approximate biological categories: angiogenesis, apoptosis, energetics, genome instability, growth suppressors, immortality, inflammation, invasion, and proliferation. We have also included the gene signature quality control metrics for the adjacent normal tissue sets in these figures as well, showing that in each case, the applicability of each signature to these datasets remains meaningful and comparable to the tumour datasets.

Briefly, sigQC, as stated above, is a protocol designed to test multiple metrics for gene signature quality on independent datasets. The radar plots summarise a total of 14 metrics that are computed by the protocol. In the following, we describe each of the metrics in turn, and for a complete description of the calculations behind each of the metrics, we refer the reader to the protocol [1]. The Relative Med. SD. metric refers to the relative median standard deviation of signature genes as compared median standard deviation of all genes. The metric $\rho_{Med.,Z-Med.}$ refers to the absolute correlation coefficient of median of signature genes and median of signature genes on the z-transformed dataset. The metrics $\rho_{Mean,PCA1}$, $\rho_{PCA1,Med.}$, and $\rho_{Mean,Med.}$ refer to the absolute correlation coefficients of mean and first principal component, first principal component and median, or mean and median, of the signature genes, respectively. The

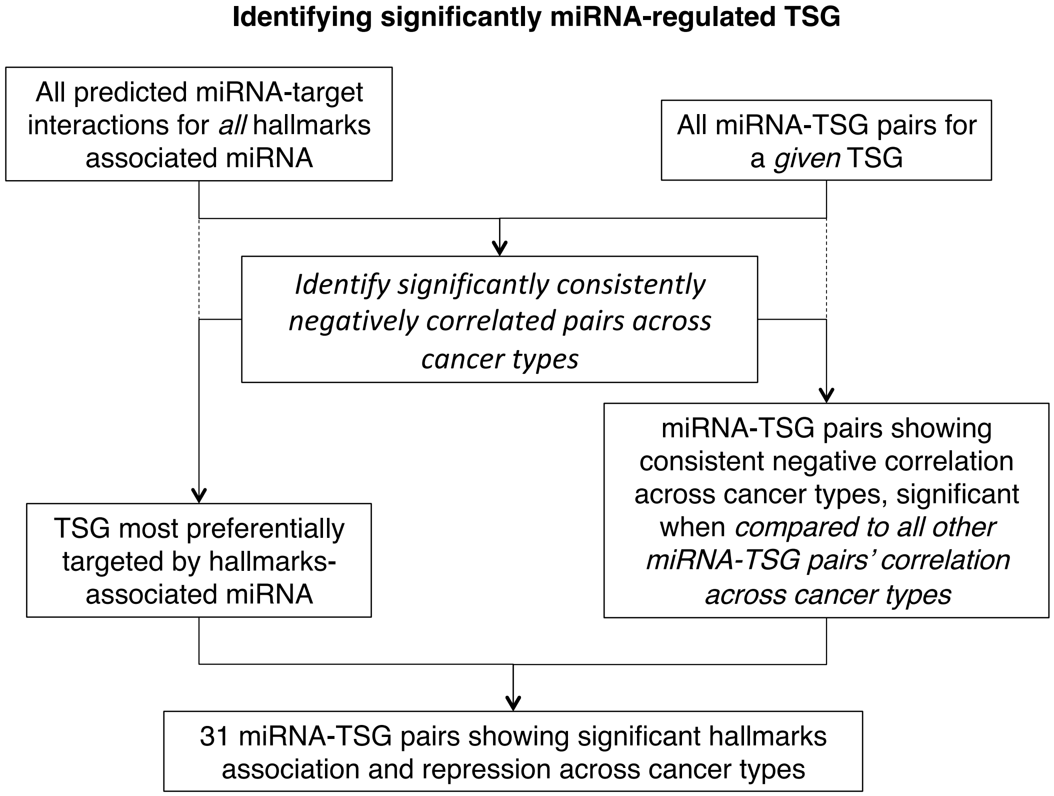

Supplementary Figure 1. Flow diagram of the methodological approach taken in the main text for identifying TSG-miRNA pairs showing significant repression across cancer types.

Autocor./Intra-sig. Corr. metric (in later versions) refers to the median of the intra-signature correlation between signature genes' expression, highlighting cohesiveness of the signature's behaviour. The metrics Prop. Expressed and Non-NA Prop give the median proportion of samples expressing signature genes above the median expression level of all genes in the dataset, or the median proportion of signature genes not reported as NA values, respectively. The metric Coef. of Var. refers to the median coefficient of variation of all signature genes, relative to the median coefficient of variation of all genes in the dataset, highlighting how well signature genes capture sample variability. The metrics $\sigma_{\geq 50\%}$, $\sigma_{\geq 25\%}$, and $\sigma_{\geq 10\%}$, report the proportion of signature genes in the top 50%, 25%, and 10% of all varying genes, respectively. The Skewness metric reports the relative skew of distribution of signature gene expression over all samples compared with skewness of expression distribution for all genes across all samples. Lastly, the σ_{PCA1} metric refers to the proportion of variance of the median signature score represented by the first principal component of signature gene expression. Taken together, these metrics provide a thorough statistical description of the properties of the signature genes' with respect to all other genes reported, highlighting whether they are expressed, variable, function as a metagene, and can be reliably summarised into a single score.

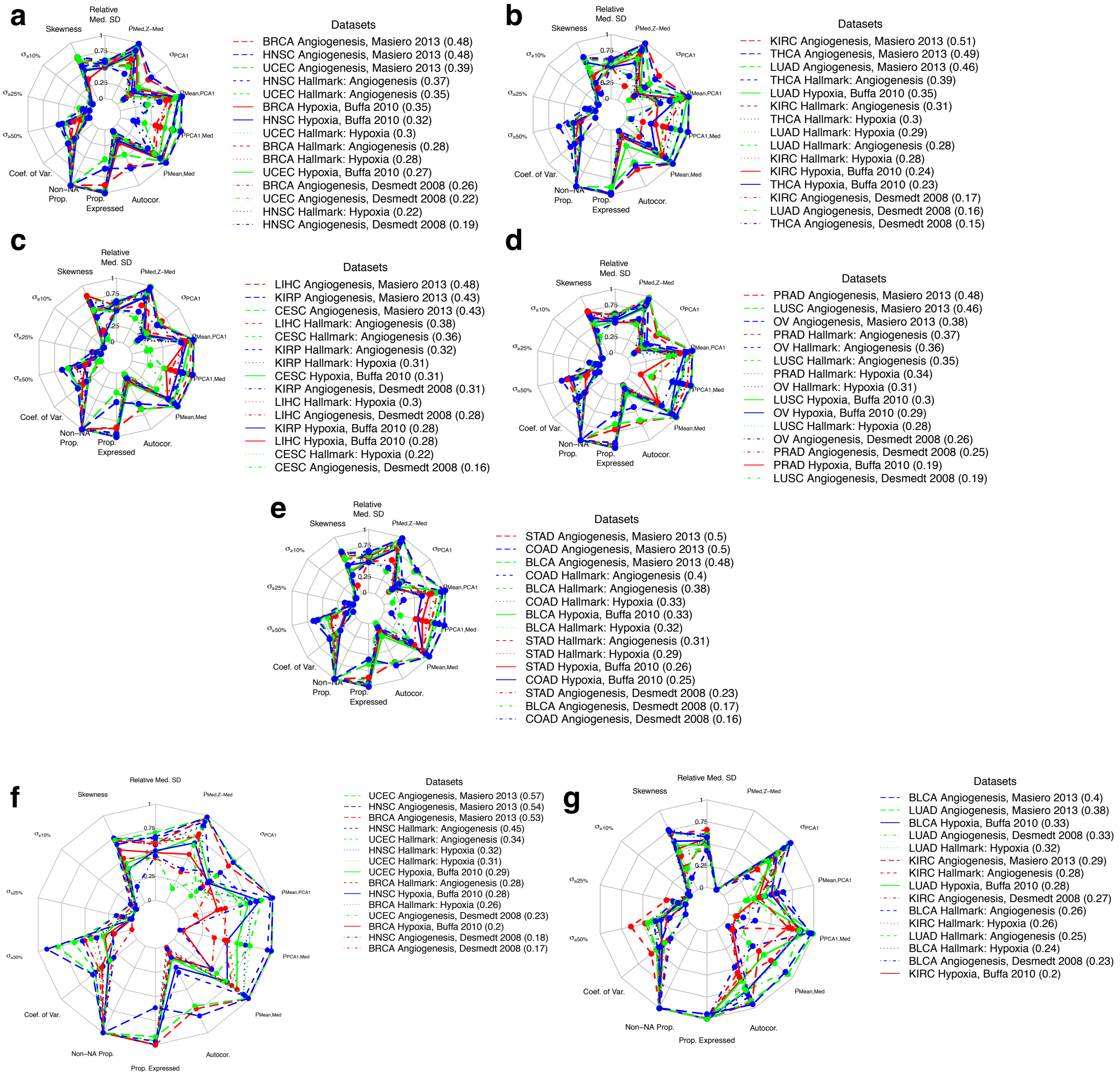

Supplementary Figure 2. sigQC radar plots for angiogenesis-related gene signatures. (**a**) Shows signatures on BRCA, UCEC, and HNSC datasets, (**b**) Shows signatures on KIRC, LUAD, and THCA datasets, (**c**) Shows signatures on LIHC, CESC, and KIRP datasets, (**d**) Shows signatures on PRAD, LUSC, and OV datasets,and (**e**) Shows signatures on STAD, BLCA, and COAD datasets. (**f**) Shows signatures on BRCA, UCEC, and HNSC adjacent normal datasets. (**g**) Shows signatures on KIRC, LUAD, and BLCA adjacent normal datasets.

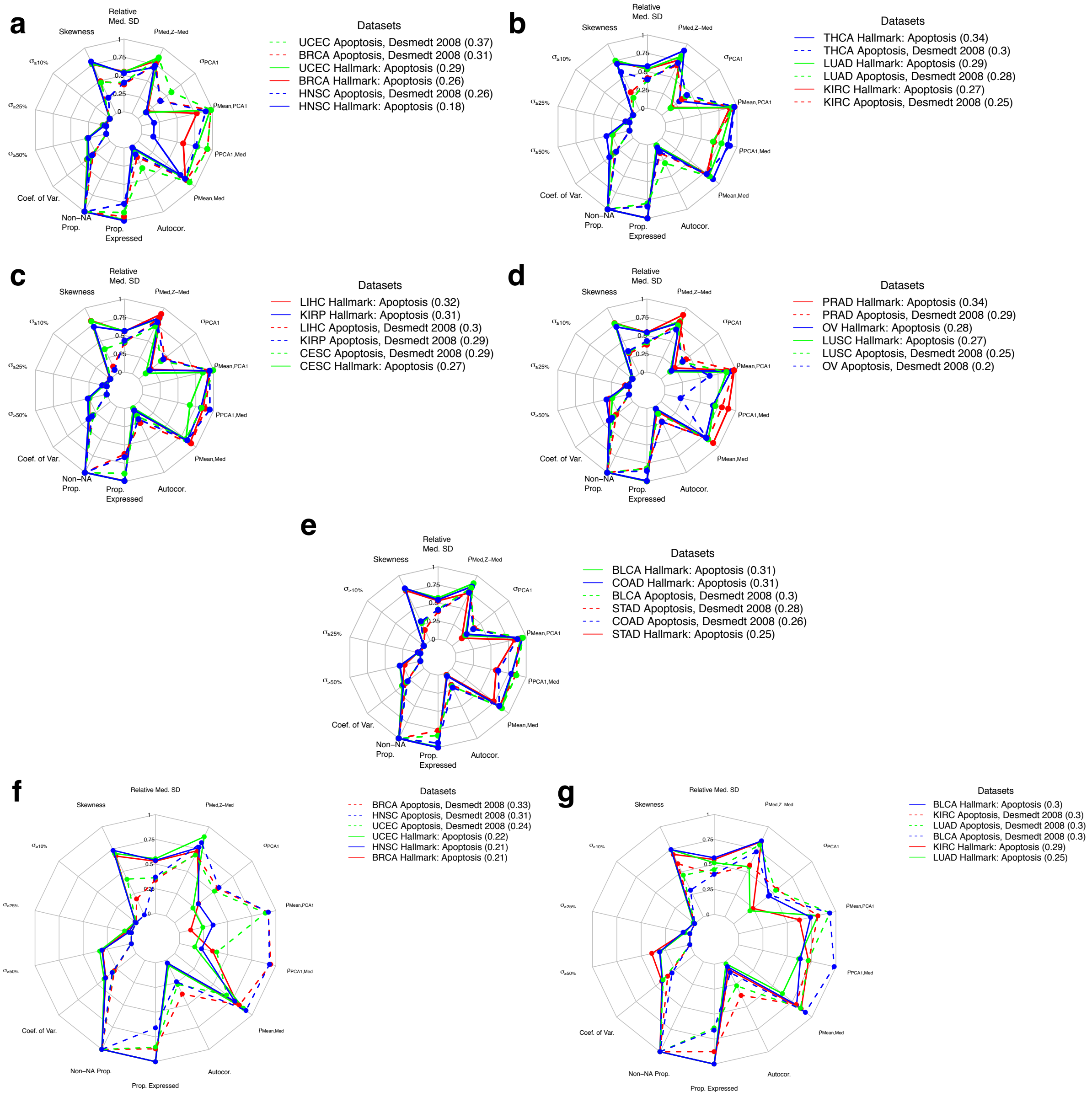

Supplementary Figure 3. sigQC radar plots for apoptosis-related gene signatures. (**a**) Shows signatures on BRCA, UCEC, and HNSC datasets, (**b**) Shows signatures on KIRC, LUAD, and THCA datasets, (**c**) Shows signatures on LIHC, CESC, and KIRP datasets, (**d**) Shows signatures on PRAD, LUSC, and OV datasets,and (**e**) Shows signatures on STAD, BLCA, and COAD datasets. (**f**) Shows signatures on BRCA, UCEC, and HNSC adjacent normal datasets. (**g**) Shows signatures on KIRC, LUAD, and BLCA adjacent normal datasets.

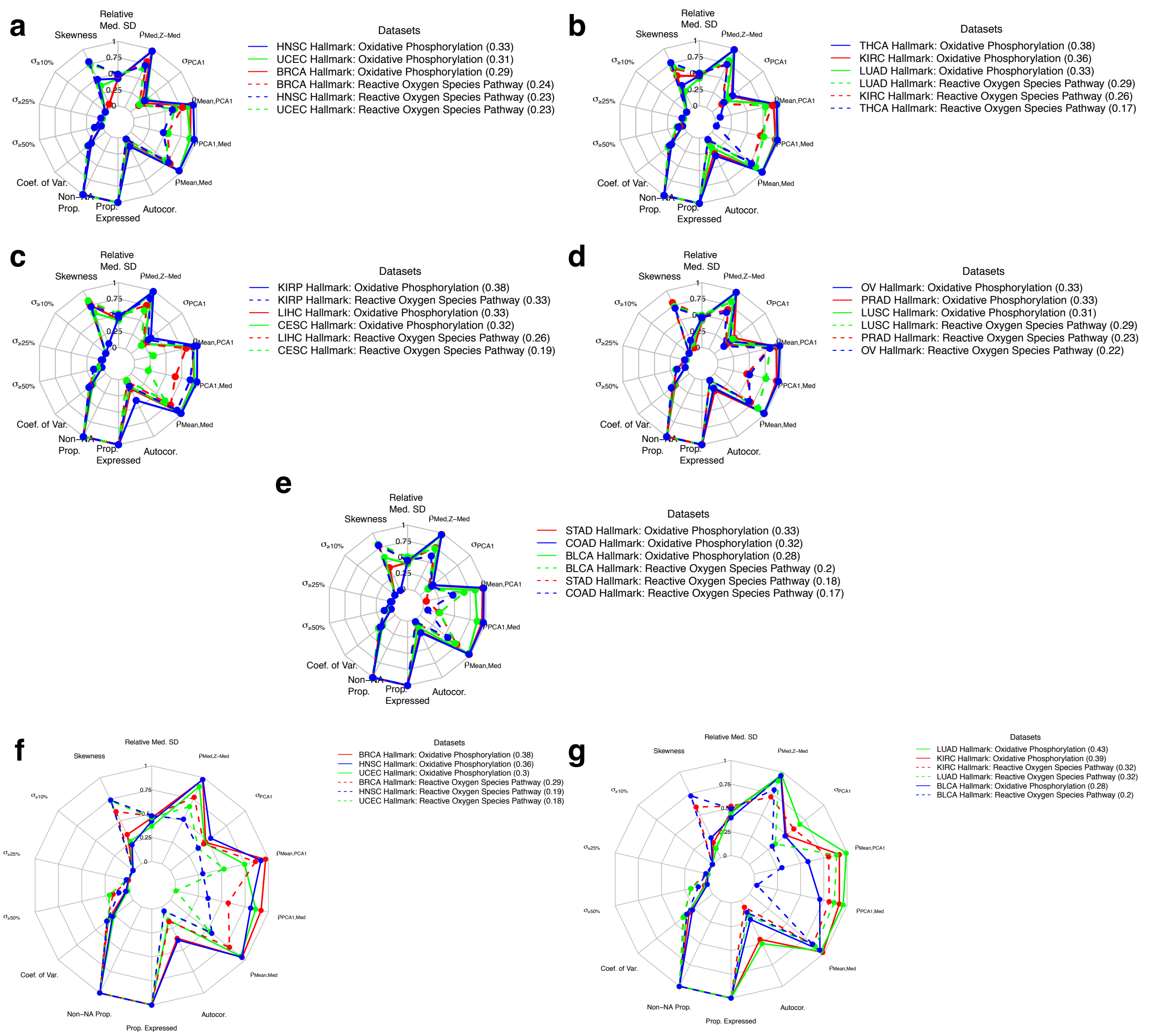

Supplementary Figure 4. sigQC radar plots for energetics-related gene signatures. (**a**) Shows signatures on BRCA, UCEC, and HNSC datasets, (**b**) Shows signatures on KIRC, LUAD, and THCA datasets, (**c**) Shows signatures on LIHC, CESC, and KIRP datasets, (**d**) Shows signatures on PRAD, LUSC, and OV datasets,and (**e**) Shows signatures on STAD, BLCA, and COAD datasets. (**f**) Shows signatures on BRCA, UCEC, and HNSC adjacent normal datasets. (**g**) Shows signatures on KIRC, LUAD, and BLCA adjacent normal datasets.

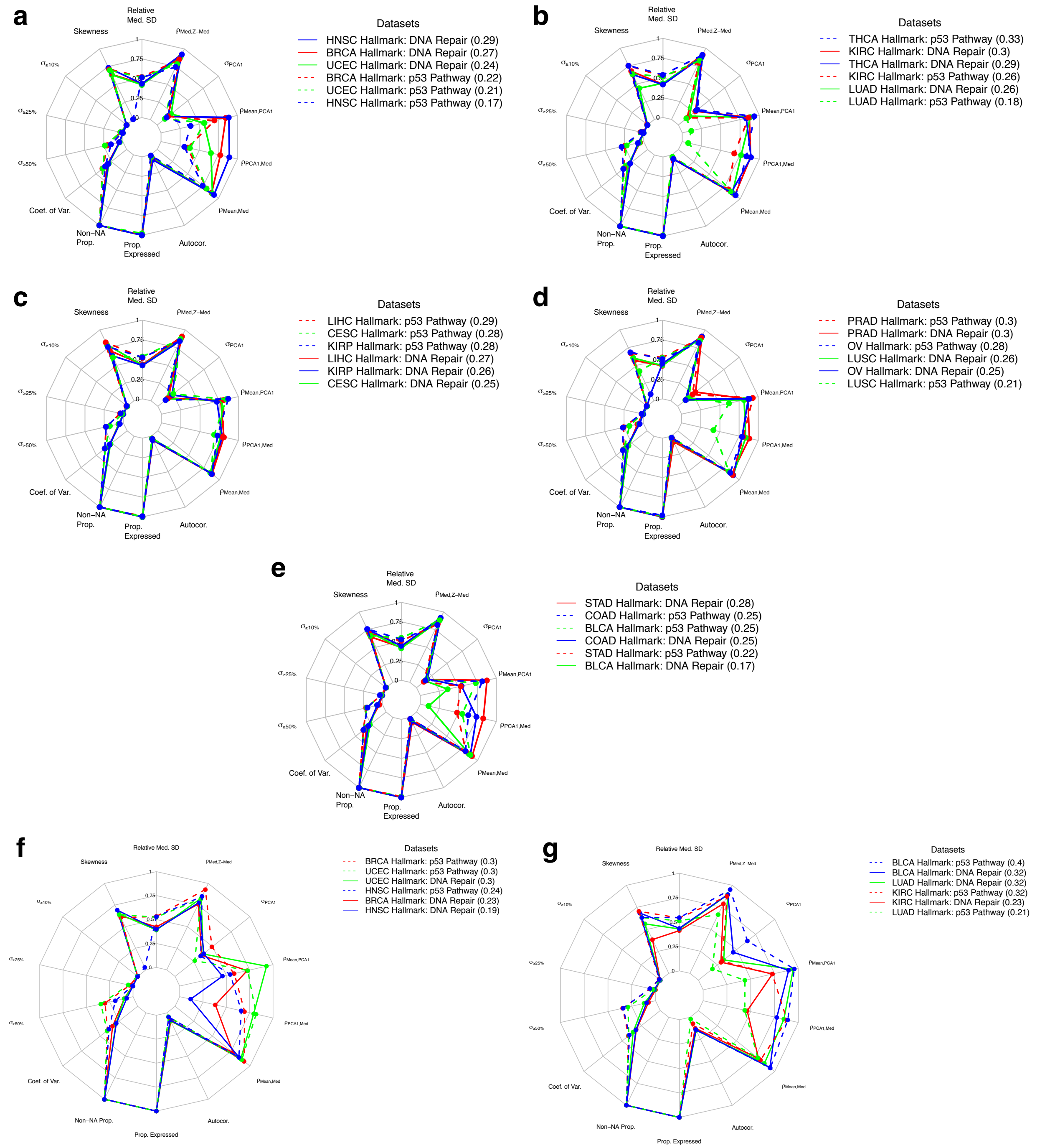

Supplementary Figure 5. sigQC radar plots for genome instability-related gene signatures. (**a**) Shows signatures on BRCA, UCEC, and HNSC datasets, (**b**) Shows signatures on KIRC, LUAD, and THCA datasets, (**c**) Shows signatures on LIHC, CESC, and KIRP datasets, (**d**) Shows signatures on PRAD, LUSC, and OV datasets,and (**e**) Shows signatures on STAD, BLCA, and COAD datasets. (**f**) Shows signatures on BRCA, UCEC, and HNSC adjacent normal datasets. (**g**) Shows signatures on KIRC, LUAD, and BLCA adjacent normal datasets.

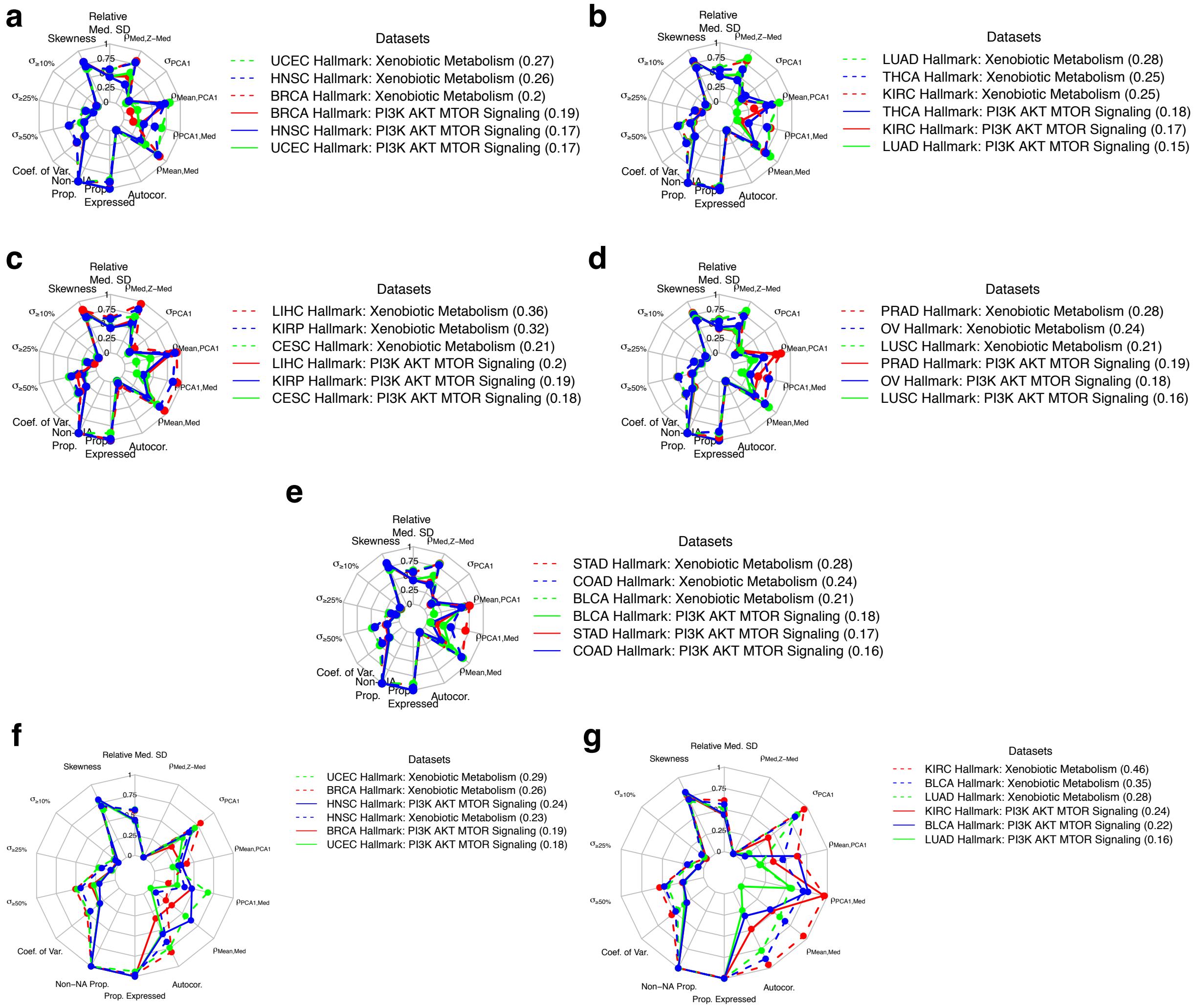

Supplementary Figure 6. sigQC radar plots for growth suppressor-related gene signatures. (**a**) Shows signatures on BRCA, UCEC, and HNSC datasets, (**b**) Shows signatures on KIRC, LUAD, and THCA datasets, (**c**) Shows signatures on LIHC, CESC, and KIRP datasets, (**d**) Shows signatures on PRAD, LUSC, and OV datasets,and (**e**) Shows signatures on STAD, BLCA, and COAD datasets. (**f**) Shows signatures on BRCA, UCEC, and HNSC adjacent normal datasets. (**g**) Shows signatures on KIRC, LUAD, and BLCA adjacent normal datasets.

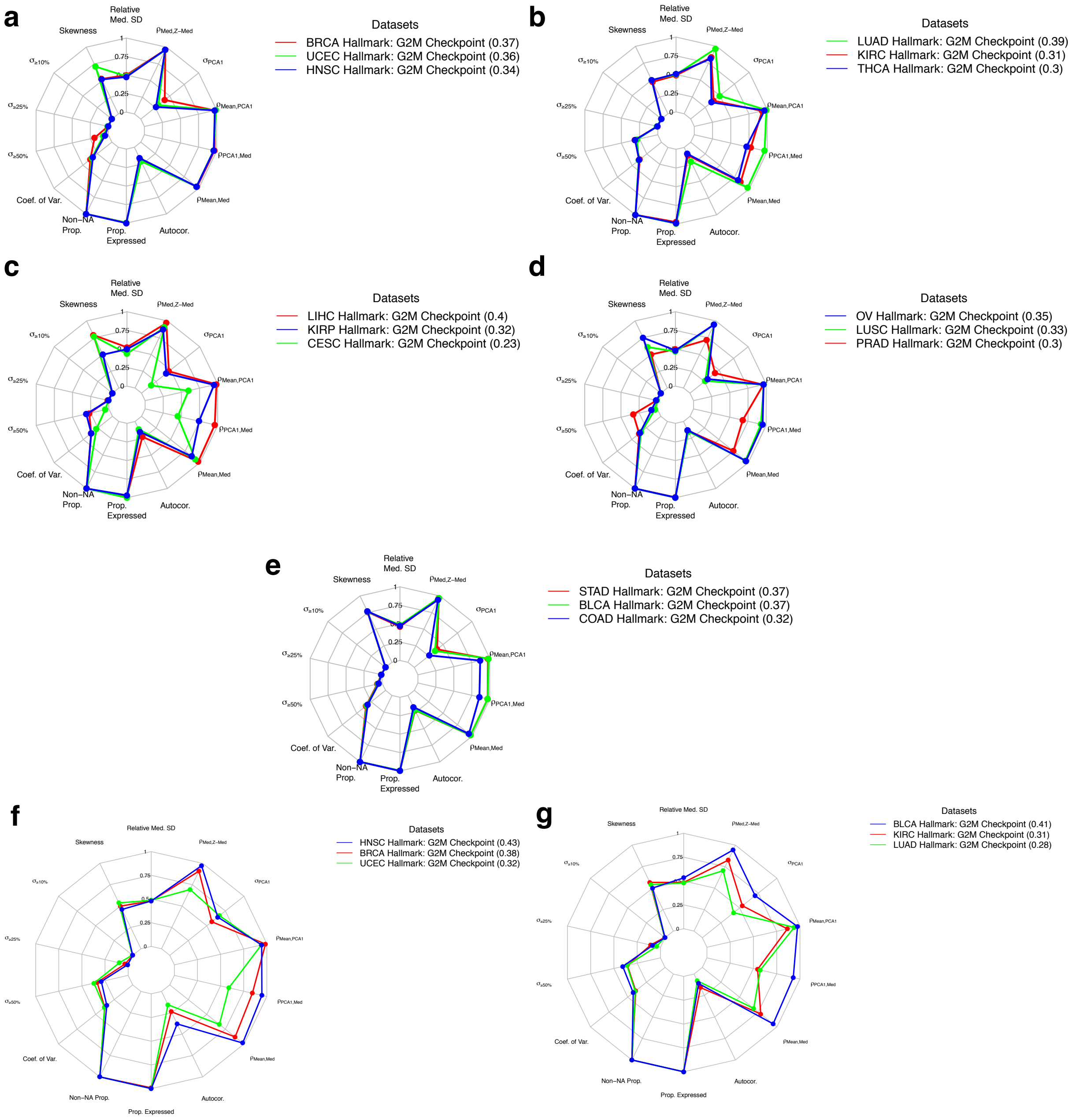

Supplementary Figure 7. sigQC radar plots for immortality-related gene signatures. (**a**) Shows signatures on BRCA, UCEC, and HNSC datasets, (**b**) Shows signatures on KIRC, LUAD, and THCA datasets, (**c**) Shows signatures on LIHC, CESC, and KIRP datasets, (**d**) Shows signatures on PRAD, LUSC, and OV datasets,and (**e**) Shows signatures on STAD, BLCA, and COAD datasets. (**f**) Shows signatures on BRCA, UCEC, and HNSC adjacent normal datasets. (**g**) Shows signatures on KIRC, LUAD, and BLCA adjacent normal datasets.

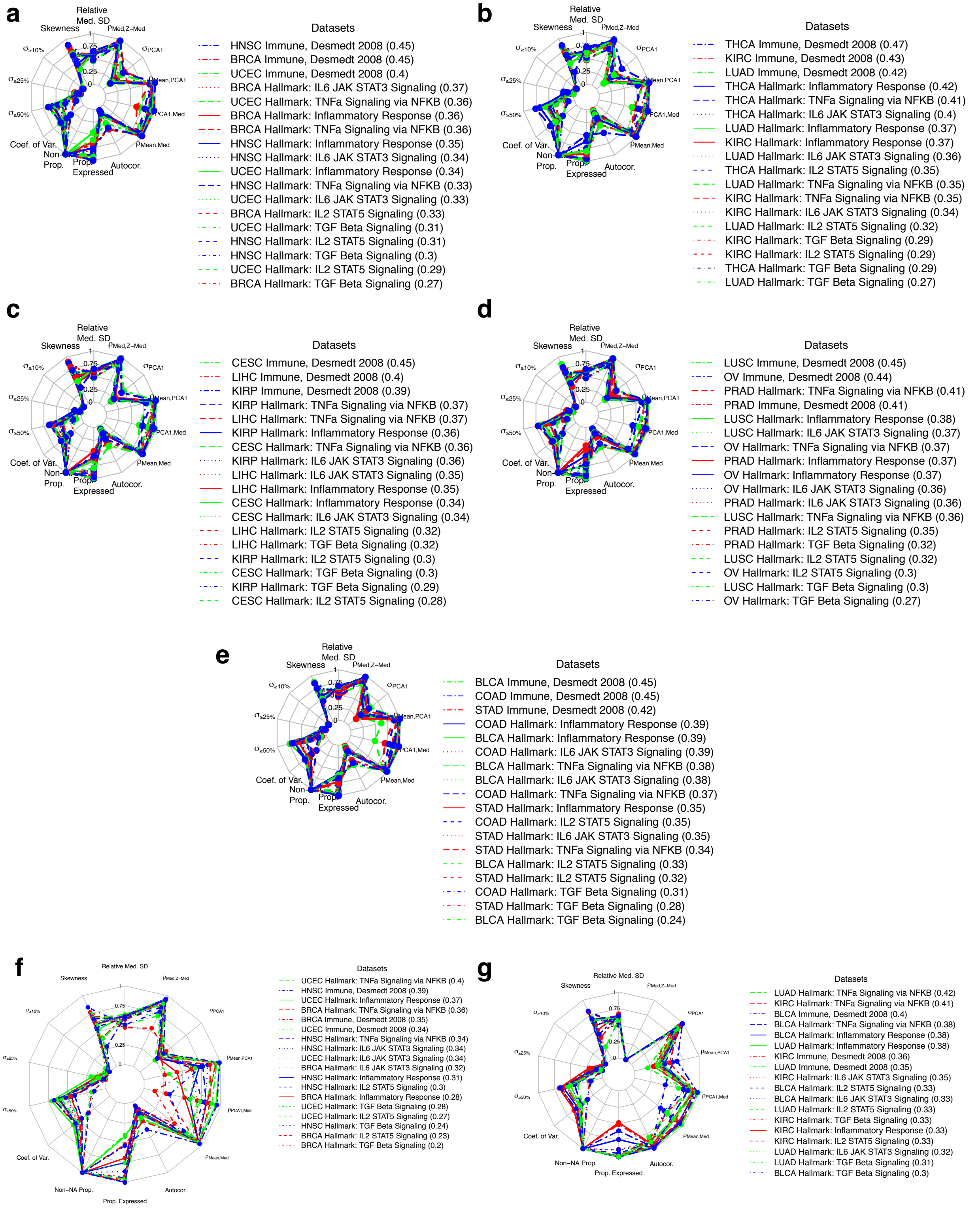

Supplementary Figure 8. sigQC radar plots for inflammation-related gene signatures. (**a**) Shows signatures on BRCA, UCEC, and HNSC datasets, (**b**) Shows signatures on KIRC, LUAD, and THCA datasets, (**c**) Shows signatures on LIHC, CESC, and KIRP datasets, (**d**) Shows signatures on PRAD, LUSC, and OV datasets,and (**e**) Shows signatures on STAD, BLCA, and COAD datasets. (**f**) Shows signatures on BRCA, UCEC, and HNSC adjacent normal datasets. (**g**) Shows signatures on KIRC, LUAD, and BLCA adjacent normal datasets.

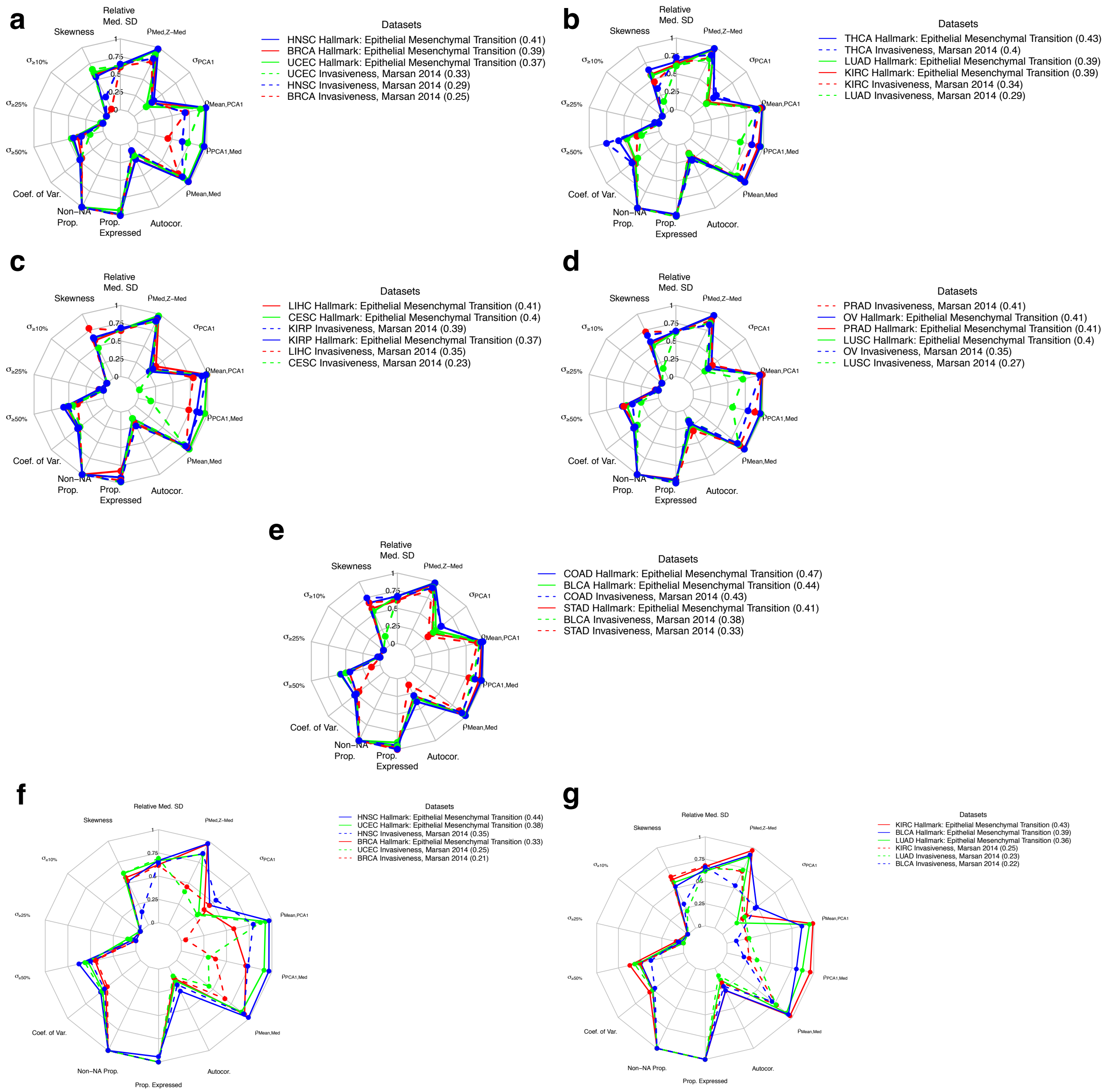

Supplementary Figure 9. sigQC radar plots for invasion-related gene signatures. (**a**) Shows signatures on BRCA, UCEC, and HNSC datasets, (**b**) Shows signatures on KIRC, LUAD, and THCA datasets, (**c**) Shows signatures on LIHC, CESC, and KIRP datasets, (**d**) Shows signatures on PRAD, LUSC, and OV datasets,and (**e**) Shows signatures on STAD, BLCA, and COAD datasets. (**f**) Shows signatures on BRCA, UCEC, and HNSC adjacent normal datasets. (**g**) Shows signatures on KIRC, LUAD, and BLCA adjacent normal datasets.

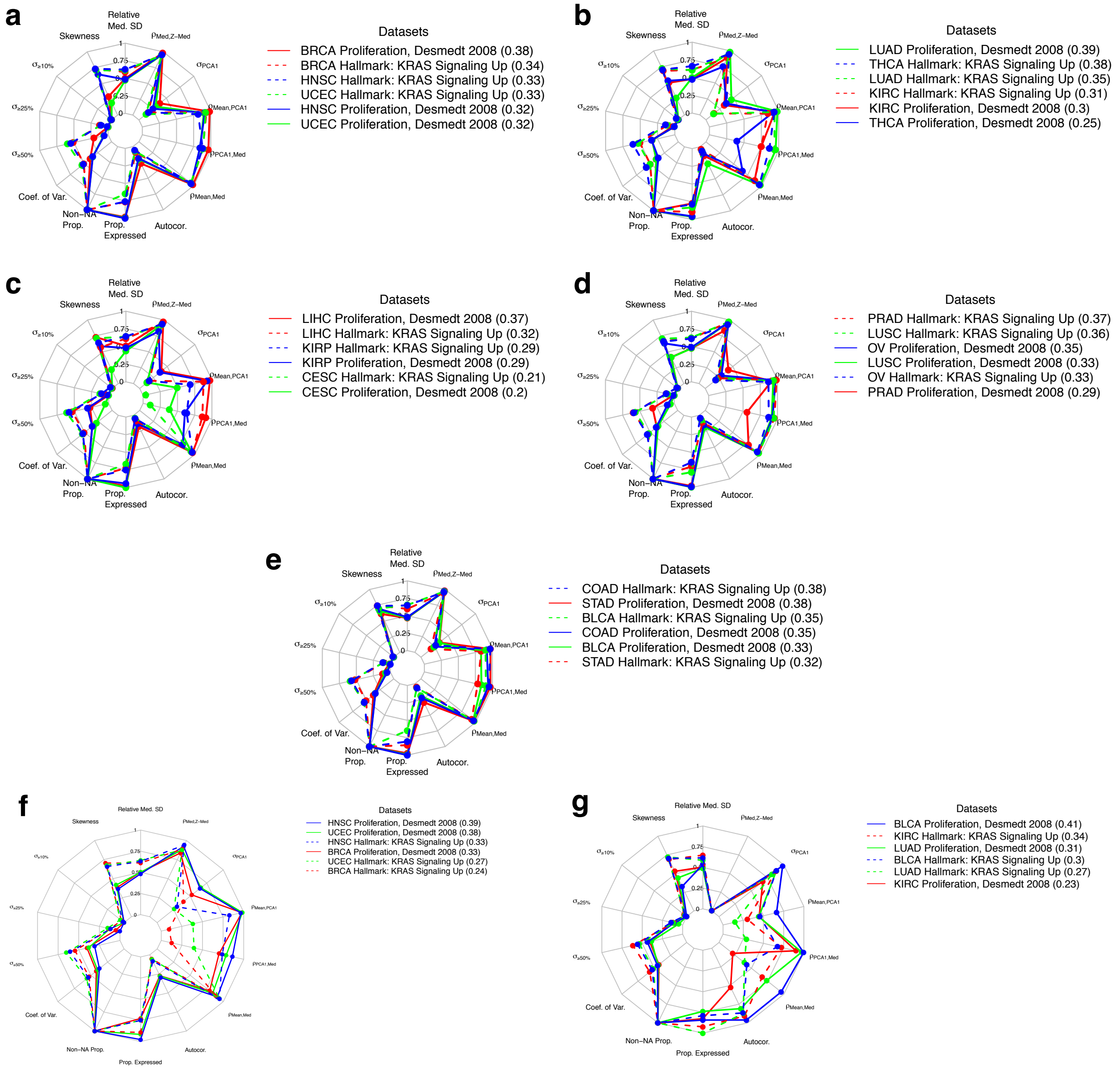

Supplementary Figure 10. sigQC radar plots for proliferation-related gene signatures. (**a**) Shows signatures on BRCA, UCEC, and HNSC datasets, (**b**) Shows signatures on KIRC, LUAD, and THCA datasets, (**c**) Shows signatures on LIHC, CESC, and KIRP datasets, (**d**) Shows signatures on PRAD, LUSC, and OV datasets,and (**e**) Shows signatures on STAD, BLCA, and COAD datasets. (**f**) Shows signatures on BRCA, UCEC, and HNSC adjacent normal datasets. (**g**) Shows signatures on KIRC, LUAD, and BLCA adjacent normal datasets.

Supplementary Note 3. Tables of positively and negatively-associated hallmarks miRNA

Tables providing the miRNA found to be significantly positively associated with each gene signature, and their corresponding rank product statistic p-values (Bonferroni corrected p-value < 0.05) and PFP values (false positive rate < 0.05) can be found in the supplementary .zip file in the signature_associated_miRNA / miRNA_up subfolder. Likewise, the downregulated miRNA and associated tables can be found in the signature_associated_miRNA / miRNA_down subfolder.

Supplementary Note 4. miRNA network for all downregulated miRNA

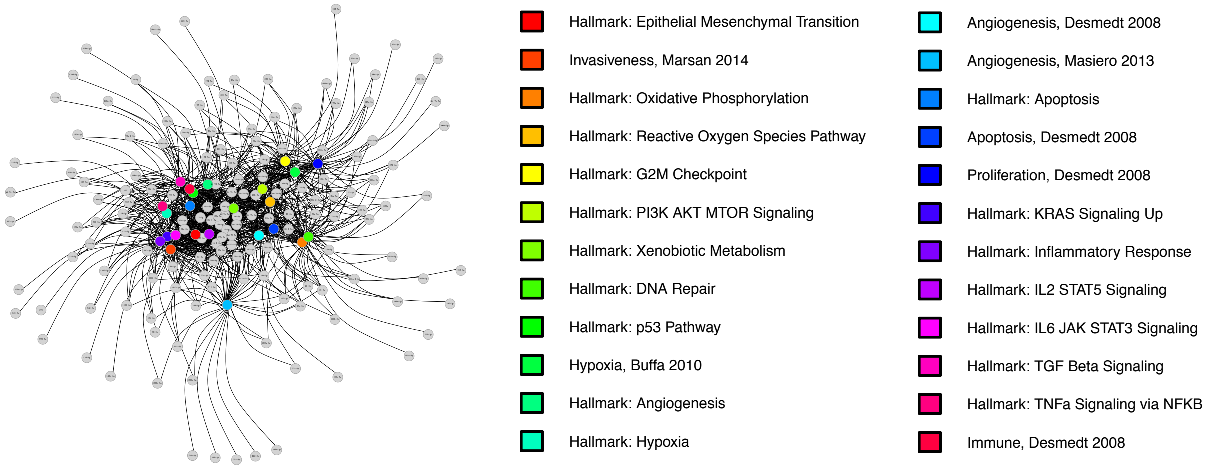

Supplementary Figure 11. Map of hallmarks and their negatively associated miRNA, analogous to Figure 1d showing strong interconnectivity between distinct molecular signatures.

Supplementary Note 5a. Concordance with validation on Metabric tumour dataset

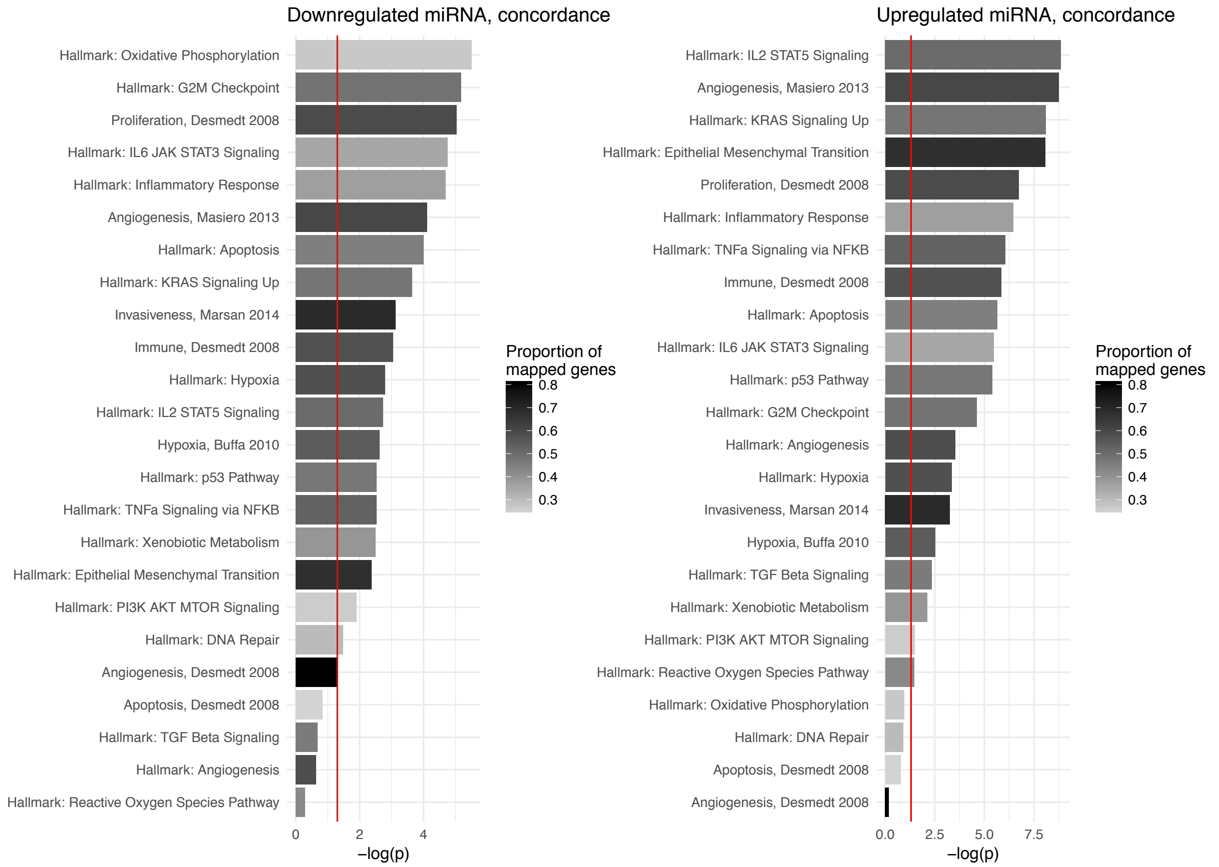

Supplementary Figure 12. Results of signature-associated miRNA validation on the Metabric dataset. Bar chart depicts -log of the p-value for the Fisher exact test examining the overlap of the signature associated miRNA identified as positive (right) or negative (left) predictors of gene signature score in the Metabric breast cancer dataset as compared to those identified by an analogous approach in the TCGA pan-cancer dataset. Vertical red bar highlights the significance cutoff of $p = 0.05$. Bars are coloured as a function of proportion of signature genes mapped to mRNA probes present within the Metabric dataset.

Supplementary Note 5b. Concordance of results when breast cancer removed from analysis

In addition to the above validation on the Metabric cohort, we test the effects of repeating our analysis with breast cancers removed, to identify whether the miRNA we have identified are significantly associated with breast cancer alone as this comprises the majority of patients. As can be seen in Supplementary figure 13, this is not the case. There is a very strong overlap with the positively associated miRNA (Up) with each signature, as well as with the negatively associated miRNA (Down) with each signature.

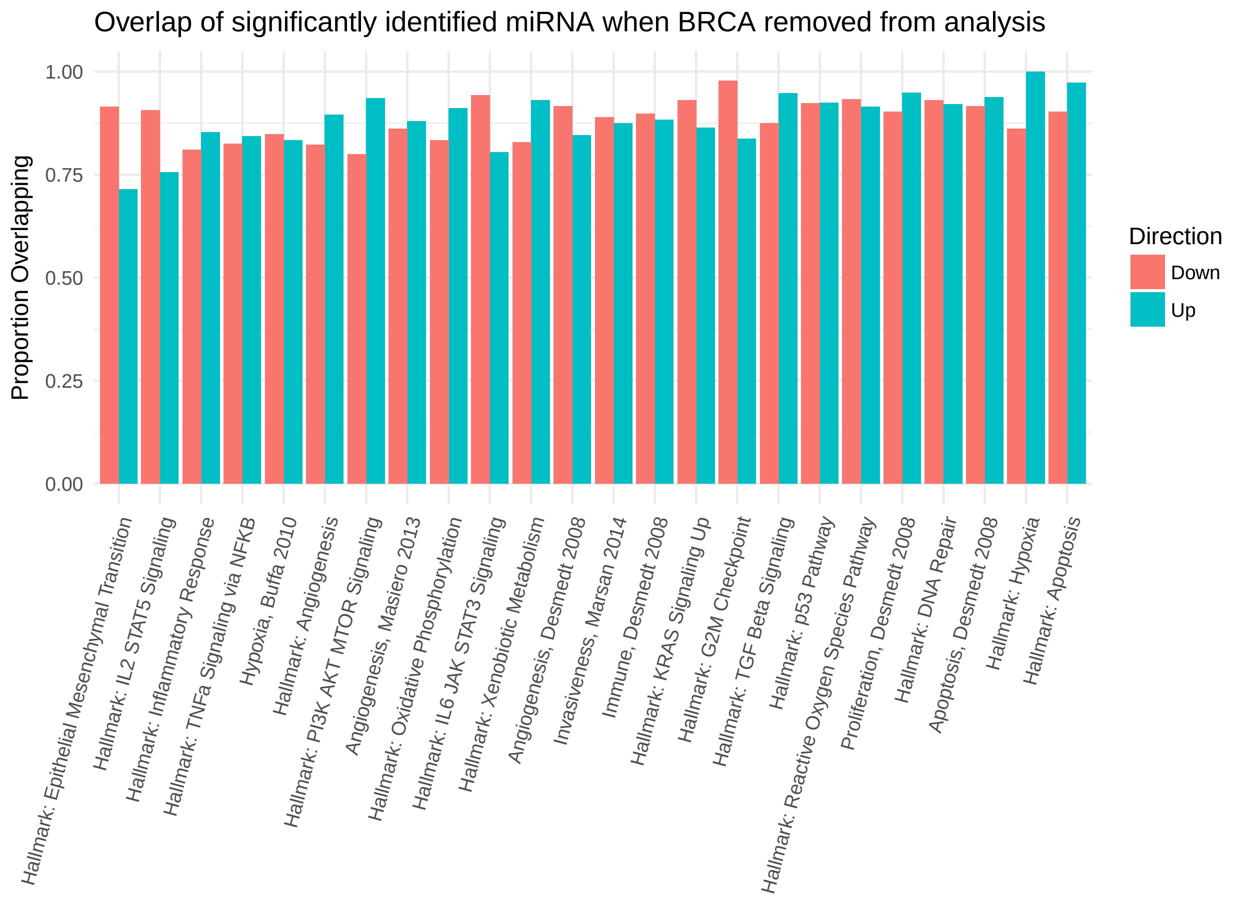

Supplementary Figure 13. Results of signature-associated miRNA overlap when breast cancers are removed from our analysis. Bar chart depicts proportion of overlapping positively-associated (Up) miRNA with each gene signature and negatively-associated (Down) miRNA with each gene signature. Results show strong concordance when breast tumours are removed, suggesting robustness of our analysis to this potential confounder.

Supplementary Note 5c. Concordance of results when using only matched tumour-normal pairs

In order to study the robustness of our results to smaller sample sizes, especially with respect to deriving an analogous miRNA-gene signature association network in normal tissues, where we used a smaller number of samples, we performed the following analysis. We first identified the miRNA as hallmarks associated (either up or down), in three groups of samples: all tumours - group A, a reduced subset of tumour samples (only those with matched normal samples) - group B, and all normal samples - group C. For each signature, we then analysed the overlap between the miRNA identified as consistently either up or down among groups A and B (comparator tumour in Supplementary figure 14), or those among groups B and C (comparator normal in Supplementary figure 14). In nearly every case, the degree of overlap between the miRNA from even the reduced set of tumour samples (just those with matched normals), is greater than the same overlap for the miRNA identified from normal samples. This underscores our use of a smaller threshold for including normal tissue types in our study, as we are able to observe a measurable

difference among miRNA identified as hallmarks-associated across signatures, with even at least 20 samples.

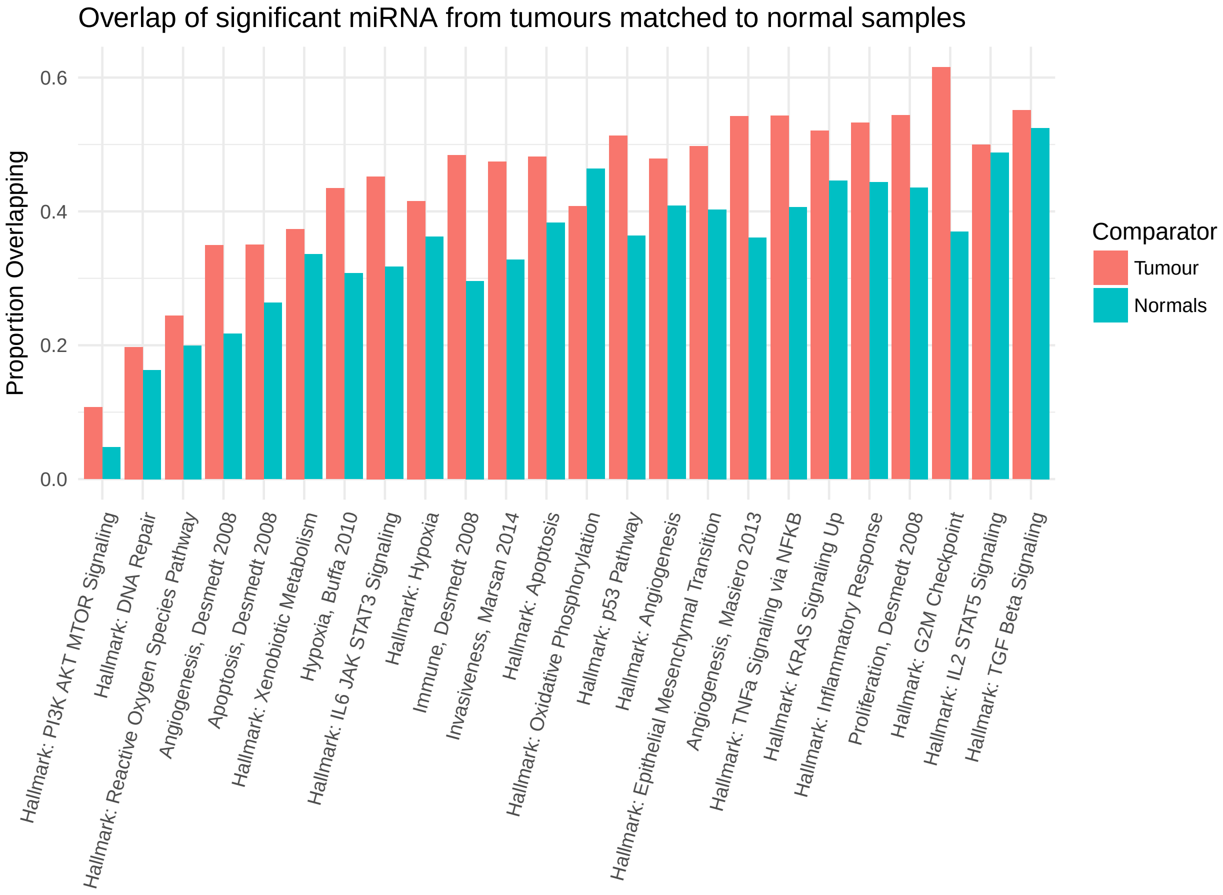

Supplementary Figure 14. Results of signature-associated miRNA overlap when only matched tumour-normal pairs of samples are considered in analysis. Results show greater concordance with tumour-associated miRNA than with normal tissue miRNA, suggesting our reduced cutoff of at least 20 samples for including normal tissues is sufficient to detect differences between the miRNA associated to each gene signature between normal and tumour tissues.

Supplementary Note 5d. Context-dependency of miRNA in breast cancer subtypes

In breast cancer, where subtypes have been established and we had larger sample size than for other cohorts, we investigated the sub-type specific context-dependence of miRNA associated to the hallmarks of cancer. However, this analysis would require much larger sample size, hence the results must be considered with caution. We considered the basal breast cancer subtype (N=83) and luminal B breast cancer subtypes (N=95), as defined for the TCGA breast cancer dataset. In a method analogous to that presented for all previous analysis, we fit, using penalised linear regression and ten fold cross-validation, a linear model describing the relationship between miRNA expression to gene signature score for the 24 hallmarks signatures considered. This was done independently for the two breast cancer subtypes considered. Once the linear models were fit and coefficients for each miRNA obtained, for each miRNA, the difference

in coefficient between the two breast cancer subtypes, for each signature, was taken. These differences were then compared across the 24 signatures, and those consistently higher or lower than expected due to chance were identified using the rank product statistic. The miRNA identified through this analysis therefore represent those that are differentially associated with the hallmarks of cancer in breast cancer subtypes. As a visualisation of this context-dependence, we have plotted the ten most associated miRNA to basal breast cancers exclusively and the ten most associated to luminal B breast cancers exclusively in Supplementary figure 15.

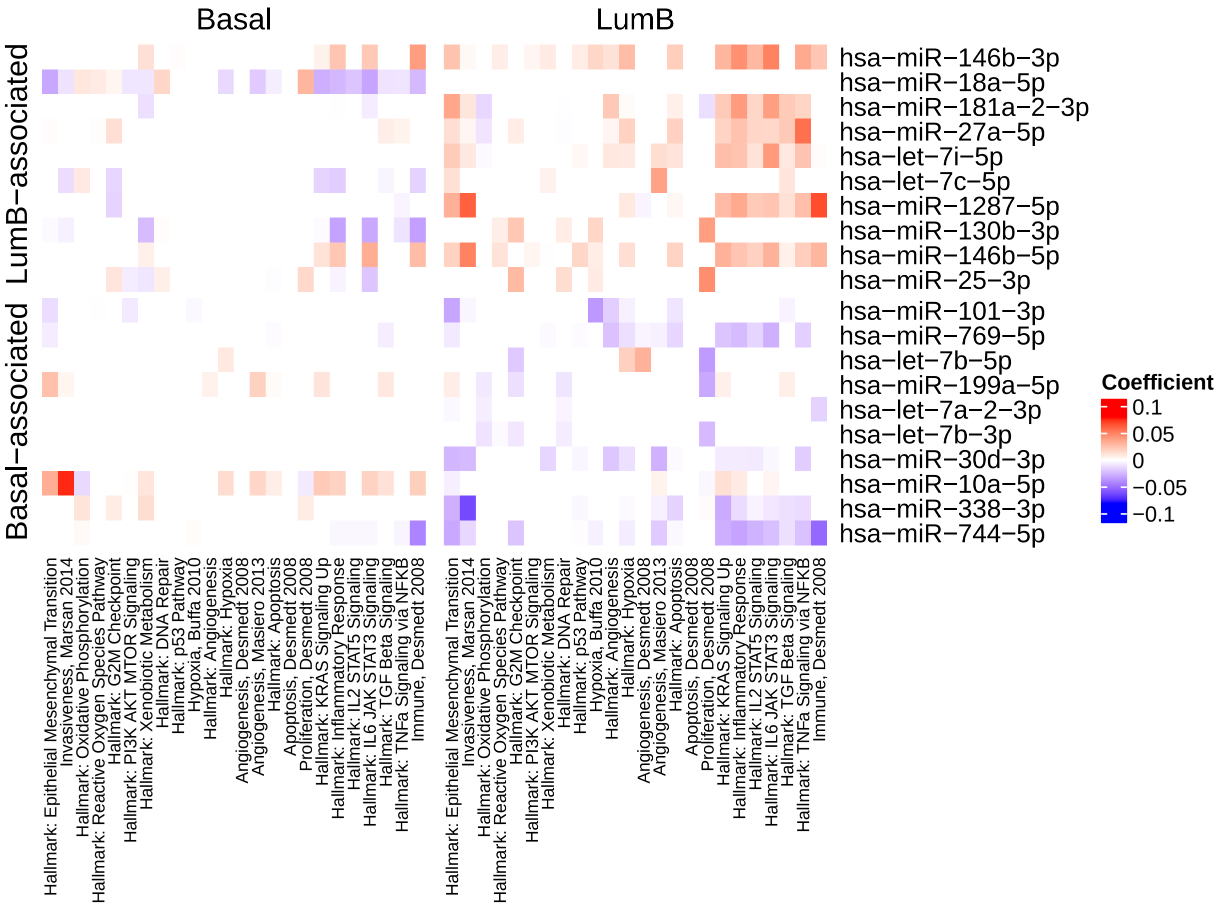

Supplementary Figure 15. Context-dependence among miRNA associated to hallmarks gene signatures between basal and luminal B breast cancer subtypes. Coefficient displayed represents the association of each miRNA to each gene signature score among either dataset.

Supplementary Note 6. Analysis of miRNA families

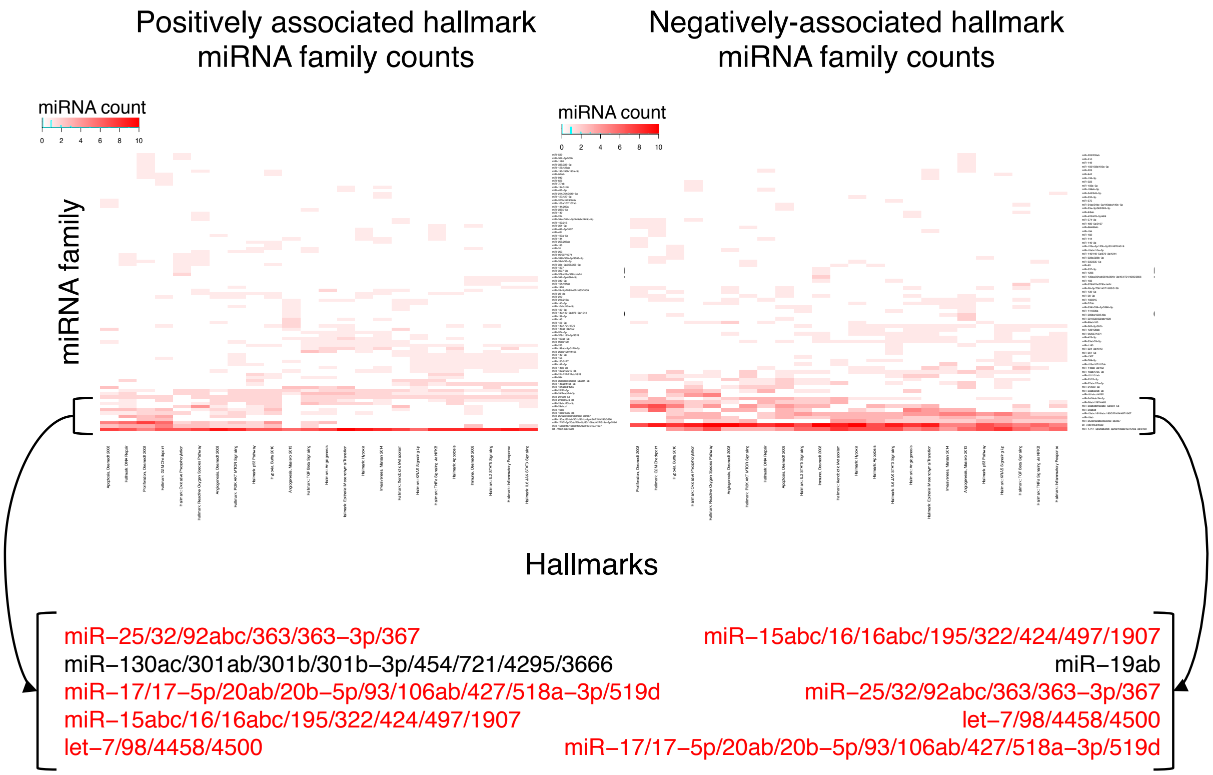

Supplementary Figure 16. Heatmaps of counts of miRNA positively (left) and negatively (right) associated with each miRNA family (rows), aggregated by gene signatures (columns). Top 5 rows from both heatmaps are enlarged, with common entries highlighted in red, showing strong concordance between both the up- and down-regulated miRNA families across signatures and cancer types.

Supplementary Note 7. TSG mutation status and associated miRNA expression

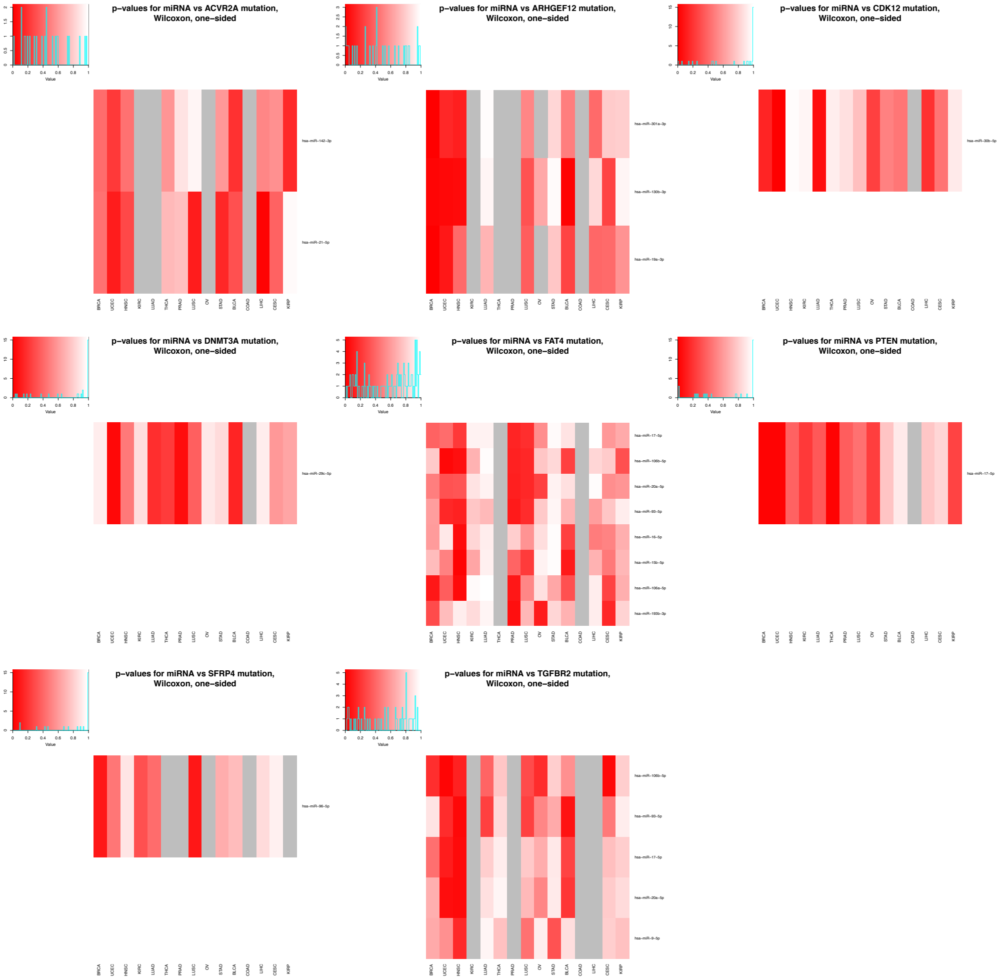

Supplementary Figure 17. Heatmap of the p values obtained for the Wilcoxon rank-sum test, one-sided, comparing TSG-associated miRNA expression for significant interactions across cancer types, for TSG mutated and wild-type cases, across tumour types (alternative hypothesis miRNA expression greater in wild-type cases). NA values are indicated in grey.

Supplementary Note 8. Rank product tables, autocorrelation heatmaps for predictors of positive and negative expression for TSG

Rank product tables, autocorrelation heatmaps for predictors of positive and negative expression for each of the 8 TSG considered can be found in the subfolder TSG, then organised by gene, in the supplementary .zip file. We have also included all of the probesets and mutation types considered for each of the tumour suppressor genes, as listed in Supplementary tables 1 and 2, below.

In addition, the ensuing pages contain the figures summarising our analysis for the evidence of exclusive regulation of TSG by miRNA, when compared with miRNA, methylation, copy number, and mutation. Briefly, as described in our methods, and in Figure 3 of the main text, we use a linear modelling scheme encompassing each of the variables for methylation levels, miRNA levels, mutation occurrence, and copy number as predictors of TSG mRNA expression across cancer types.

Here, we describe an example of how each of the results in the further supplementary figures was interpreted. We contrast the results we have obtained for two opposing cases; that of ACVR2A in stomach adenocarcinoma, which does not show evidence of exclusivity in regulation, and PTEN in bladder cancer, which does show strong evidence for exclusivity. First, recall that these two TSG are among a set of 8 TSG which appeared to have evidence of miRNA-mediated regulation across cancer types. Having obtained this set of genes, our next step was to determine whether the regulation by miRNA was occurring in the absence or presence of other modes of regulation, such as methylation, copy number aberration, or mutation. As such, we created the linear modelling paradigm as described above and in the methods section. Aggregating the coefficients obtained from the linear model across cancer types, we were able to gain an understanding of how each mode of regulation affected TSG mRNA levels, and which were important relative to the others, as positive and negative regulators. Thus, the negative coefficients in the linear model defined a set of miRNA and methylation probes, as well as the impact of mutations that would function to reduce the level of TSG mRNA.

With these lists of miRNA, methylation probes, and mutation types that we had shown to negatively associate with TSG mRNA levels across cancer types, we next wanted to determine how they co-occurred with each other, leading to the analysis for co-correlation between methylation probe expression levels, miRNA expression, and the variables for mutation occurrence. As contrasted in the sample case considered in Supplementary figure 18a below, distinct patterns emerged in some cases, such as PTEN in BLCA, suggesting that methylation probes were co-expressed with each other, and rarely with miRNA, and vice versa, thus leading to the hypothesis of exclusivity of regulation. In contrast, the case of ACVR2A in STAD shows no such separation in Supplementary figure 18a, with many significantly positively correlated miRNA and methylation probes, and no clear exclusivity.

To test this hypothesis rigorously, we devised a resampling-based approach to examine how significant the exclusivity of the regulation of each tumour suppressor gene was. By resampling sets of miRNA and methylation probes, and examining how exclusive the regulation was in each of these cases, we were able to determine the significance of how exclusively occurring these regulators were in our cases of interest, as depicted in Figure 4 in the main text, and summarised in Supplementary figure 18b below for our example case. This shows how, as expected, the exclusivity of regulation observed for ACVR2A in STAD is not statistically significant, but it is highly significant in the case of PTEN in BLCA.

After we had identified the significance of the exclusivity of regulation of each TSG by each mode of regulation, we next defined subgroups of samples for each mode of regulation, either taking the median level of the regulating miRNA, regulating methylation probes, or the presence of a mutation to define subgroups. We examined the relationship of each of these subgroups to the level of TSG expression, showing that, in some cases, the effect of each of these modes of regulation was indeed significant and important in reducing the level of TSG expression. We summarise this for our example cases in Supplementary figure 18c, below,

where we observe how the expression of ACVR2A in STAD and PTEN in BLCA changes in association with the subgroups of patients defined by the presence or absence of increased methylation, miRNA, or mutation. These results highlight the phenotypic differences between these genomically-distinct subgroups. Further analysis described in Supplementary Note 10 describes how a differential expression analysis was done to further compare transcriptomic similarities and differences between these subgroups as well.

Supplementary Table 1. Probe sets for methylation of each tumour suppressor gene considered in analysis. Data used are from TCGA methylation studies across cancer types considered.

Gene	Methylation probes
PTEN	cg01228636, cg01354923, cg02261018, cg02307823, cg03214660, cg03236184, cg03588460, cg03891929, cg04059318, cg04582473, cg04616691, cg04638773, cg04707787, cg04738091, cg04824711, cg05947570, cg06466203, cg06731059, cg06947206, cg07263825, cg07655693, cg08363193, cg08602305, cg08859916, cg08960754, cg08995089, cg09472211, cg09528884, cg09550257, cg10041390, cg10205334, cg10930218, cg12005026, cg13528847, cg13885325, cg15412736, cg16404460, cg16443434, cg16686761, cg16687447, cg17083429, cg17114151, cg17489897, cg17557106, cg18141918, cg18384060, cg18665732, cg18819818, cg18953873, cg19358349, cg19634213, cg19659388, cg20849549, cg21573601, cg22564317, cg23149470, cg23753021, cg25452974, cg26090855, cg26127345, cg27084903, cg27299538, cg27422496
ACVR2A	cg00532455, cg02093647, cg03601011, cg06120425, cg06907069, cg07526221, cg07969095, cg12968518, cg14420245, cg14425722, cg14689355, cg14926149, cg16081228, cg17174566, cg21233506, cg21579828, cg22464182, cg23727674, cg27112146
ARHGEF12	cg00395063, cg03274991, cg04919489, cg05099464, cg06767612, cg07099388, cg08318018, cg09754341, cg10407488, cg10493270, cg10738003, cg10952477, cg12819548, cg12823408, cg12851792, cg15028899, cg15143809, cg15690696, cg15892763, cg16106770, cg16757423, cg17030562, cg18567470, cg21468385, cg22276271, cg23618830, cg24566217, cg25242756, cg26098650, cg26625290, cg26681847, ch.11.2495959R
CDK12	cg00061989, cg06862673, cg08170745, cg09102835, cg10398950, cg12424509, cg12477119, cg17557704, cg20708332, cg20936107, cg21037155, cg22133495, cg26279814, cg27544759, ch.17.1008464R, ch.17.1009718F
DNMT3A	cg00050692, cg00220517, cg00277048, cg00856404, cg00886730, cg00898683, cg00912598, cg02118630, cg02208653, cg02746110, cg03314052, cg03463641, cg03766400, cg04058399, cg04436772, cg04683068, cg05516842, cg05544807, cg05652528, cg05896193, cg06112956, cg06224893, cg06748978, cg07150430, cg07720334, cg08316074, cg08485187, cg08493294, cg09986894, cg10142668, cg10239163, cg10270719, cg10525105, cg10614445, cg10616515, cg10749994, cg11343289, cg11354105, cg11779362, cg11798660, cg12066181, cg13076778, cg13344237, cg13558695, cg13828701, cg14189391, cg15150970, cg15302376, cg15843262, cg15990840, cg15998962, cg17137500, cg17207266, cg17742416, cg18889183, cg19256292, cg19489797, cg19862213, cg20303441, cg20669908, cg20702417, cg20948740, cg21598294, cg21629895, cg21708767, cg22705918, cg22731525, cg23009818, cg23042148, cg23393100, cg23569120, cg23903708, cg25044635, cg25096282, cg26470599, cg26544247, cg26803803, cg26995204, cg27369452
FAT4	cg00990763, cg03404279, cg03527919, cg04023369, cg04171487, cg04373334, cg04459504, cg05118638, cg08575049, cg08644023, cg10399929, cg10731073, cg12058185, cg12828819, cg13742182, cg15795630, cg17265829, cg17760043, cg18202623, cg22911422, cg23901852, cg25879360, cg26389756
SFRP4	cg01613122, cg01689311, cg04651042, cg05682561, cg06161814, cg08261094, cg09594069, cg10806140, cg11878069, cg12515638, cg13400306, cg14846368, cg16433922, cg18723937, cg19166347, cg20019546, cg21122375, cg22826141, cg23169784, cg23569180, cg25783719
TGFBR2	cg03256955, cg03420580, cg03630790, cg04916416, cg05074709, cg05450916, cg06270049, cg06784602, cg07285675, cg07613391, cg09417692, cg09668216, cg12419522, cg12541591, cg12926720, cg13504215, cg13724812, cg13859541, cg14910241, cg15171154, cg15270950, cg15724876, cg16299428, cg17546721, cg17786388, cg19408535, cg19482049, cg19615017, cg19995459, cg20216935, cg21814995, cg23485307, cg24321706, cg24719910, cg24952959, cg25438762, cg26376346

Supplementary Table 2. Mutation types (non-silent) considered for each tumour suppressor gene considered in analysis. Data used are from TCGA mutation studies across cancer types considered, as reported by Oncotated calls accessed from the Firebrowse data portal.

Gene	Mutation types
PTEN	Missense Mutation, Nonsense Mutation, Frame Shift Del, Frame Shift Ins, Splice Site, In Frame Del, In Frame Ins, RNA
ACVR2A	Missense Mutation, Frame Shift Del, Silent, Nonsense Mutation, Frame Shift Ins, In Frame Del, Splice Site
ARHGEF12	Missense Mutation, Nonsense Mutation, Frame Shift Del, Splice Site, Intron
CDK12	Missense Mutation, Nonsense Mutation, Frame Shift Del, Frame Shift Ins, Splice Site, In Frame Del
DNMT3A	Missense Mutation, Frame Shift Del, Splice Site, Nonsense Mutation, Frame Shift Ins
FAT4	Missense Mutation, Nonsense Mutation, Frame Shift Del, Splice Site, Frame Shift Ins, In Frame Del
SFRP4	Missense Mutation, Frame Shift Ins, Frame Shift Del, Splice Site
TGFBR2	Missense Mutation, Nonsense Mutation, In Frame Del, Frame Shift Del, Splice Site, Frame Shift Ins

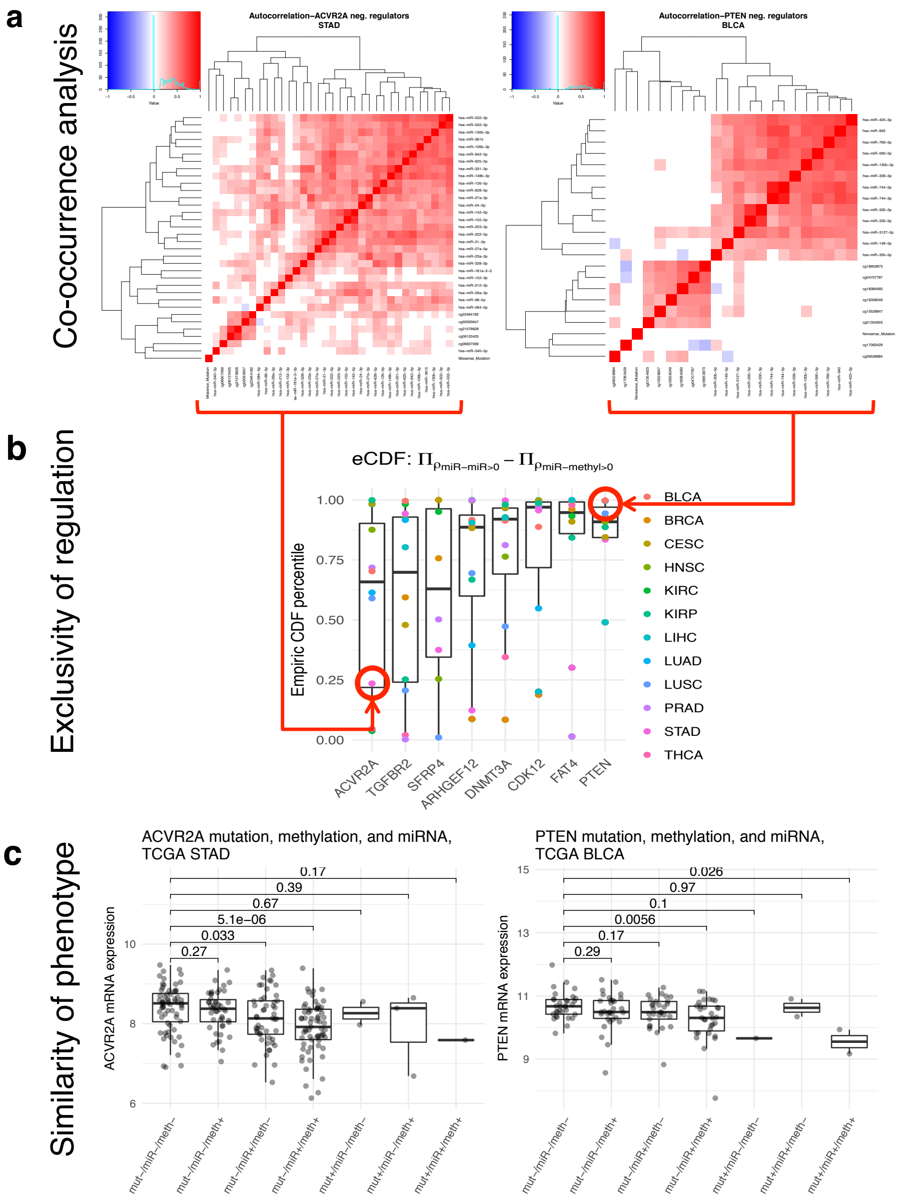

Supplementary Figure 18. Sample of analysis done to determine exclusivity of TSG regulation. Example shown is for the two differing cases of ACVR2A in STAD (left) and PTEN in BLCA (right). (**a**) Shows the heatmaps obtained for the co-correlation between the mutation occurrence, expression of methylation probes, and miRNA negatively regulating each gene, showing the visual differences in the exclusivity of the regulation of these genes. (**b**) Depicts the significance of the exclusivity observed, as computed by a resampling-based approach, highlighted with red circles for ACVR2A in STAD and PTEN in BLCA. (**c**) Depicts how the expression of the TSG of interest (ACVR2A or PTEN) varied between subgroups defined by methylation high or low, miRNA expression high or low, or mutation present or not.

Supplementary Note 9. Autocorrelation heatmaps for negative regulators of TSG

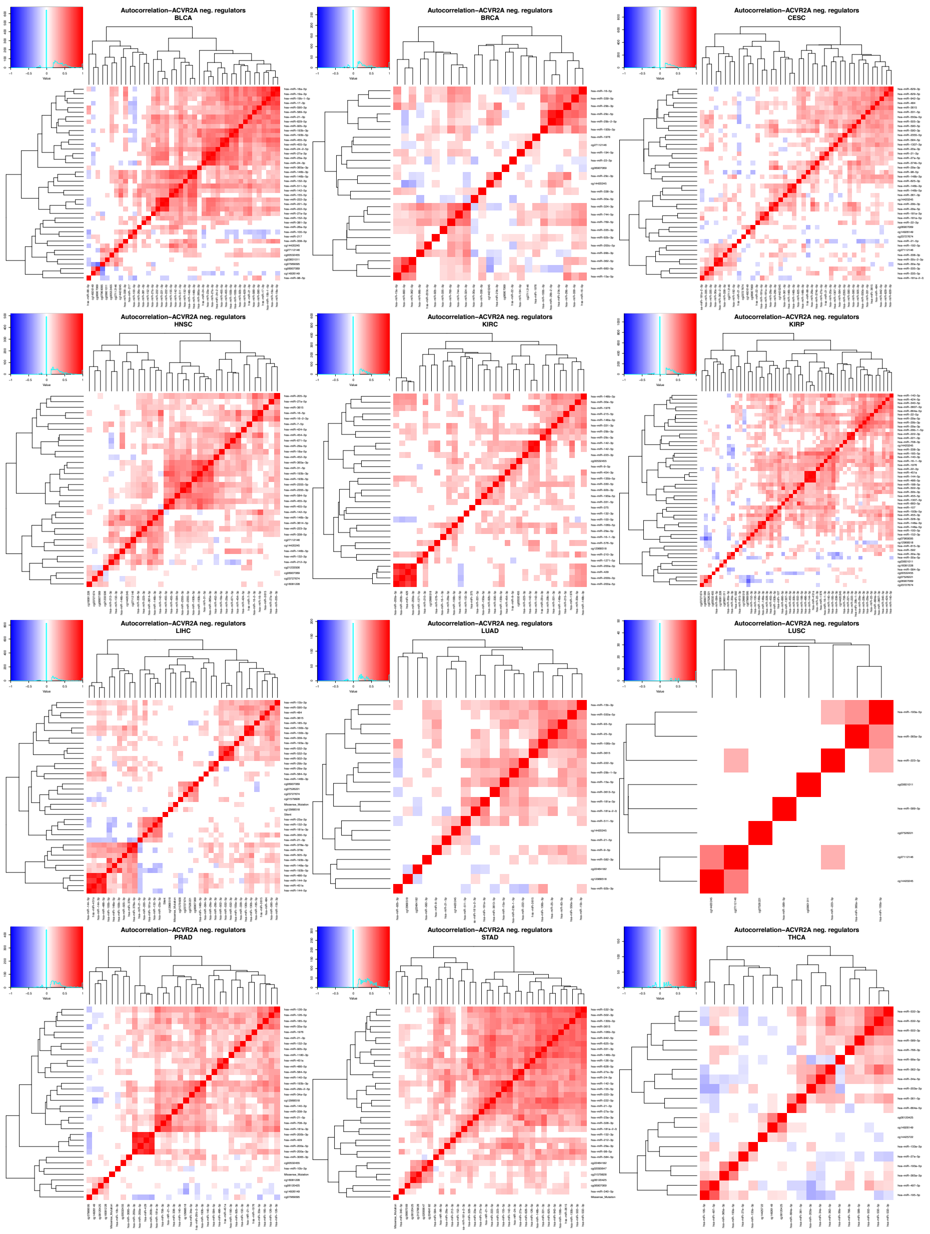

Supplementary Figure 19. Autocorrelation heatmap for the expression of the identified negative regulators of ACVR2A, across the 12 cancer types considered.

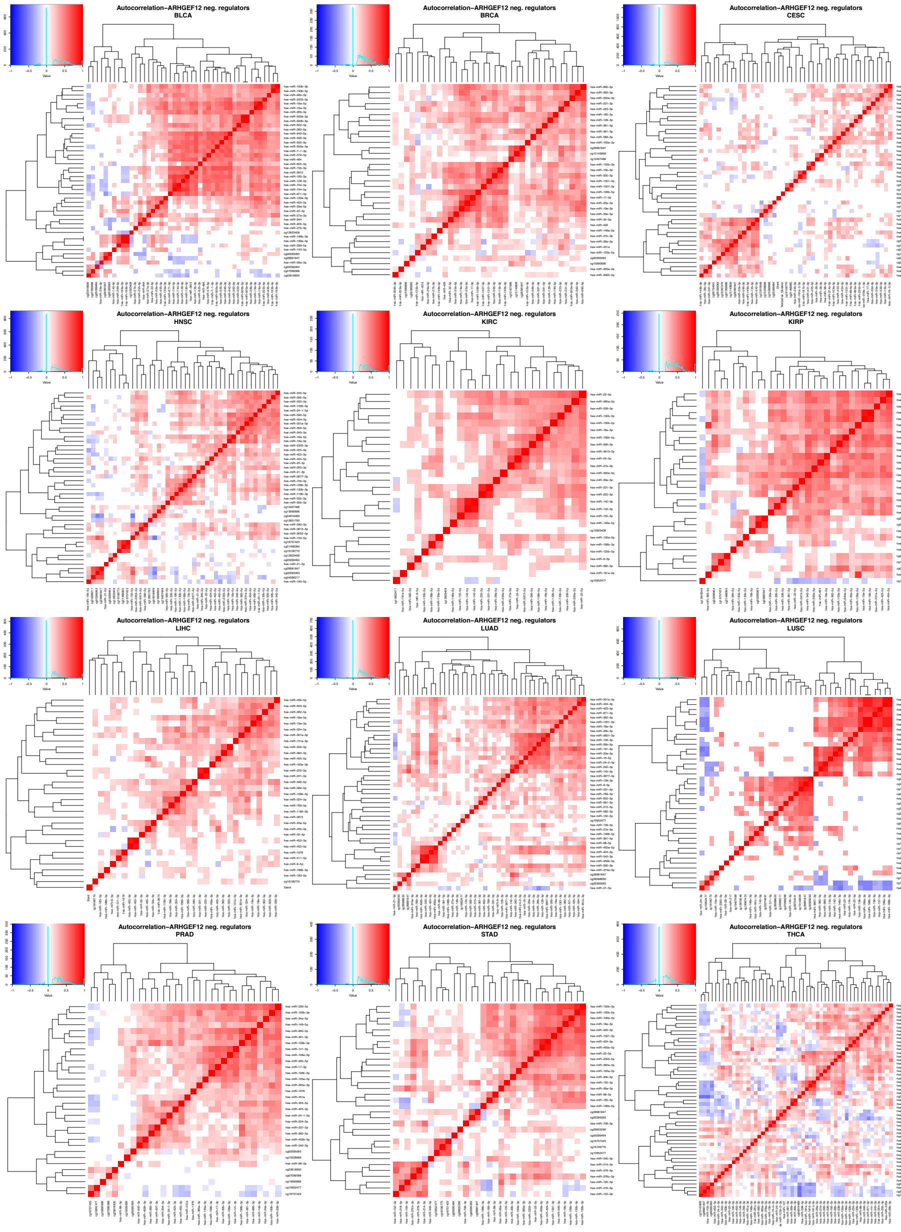

Supplementary Figure 20. Autocorrelation heatmap for the expression of the identified negative regulators of ARHGEF12, across the 12 cancer types considered.

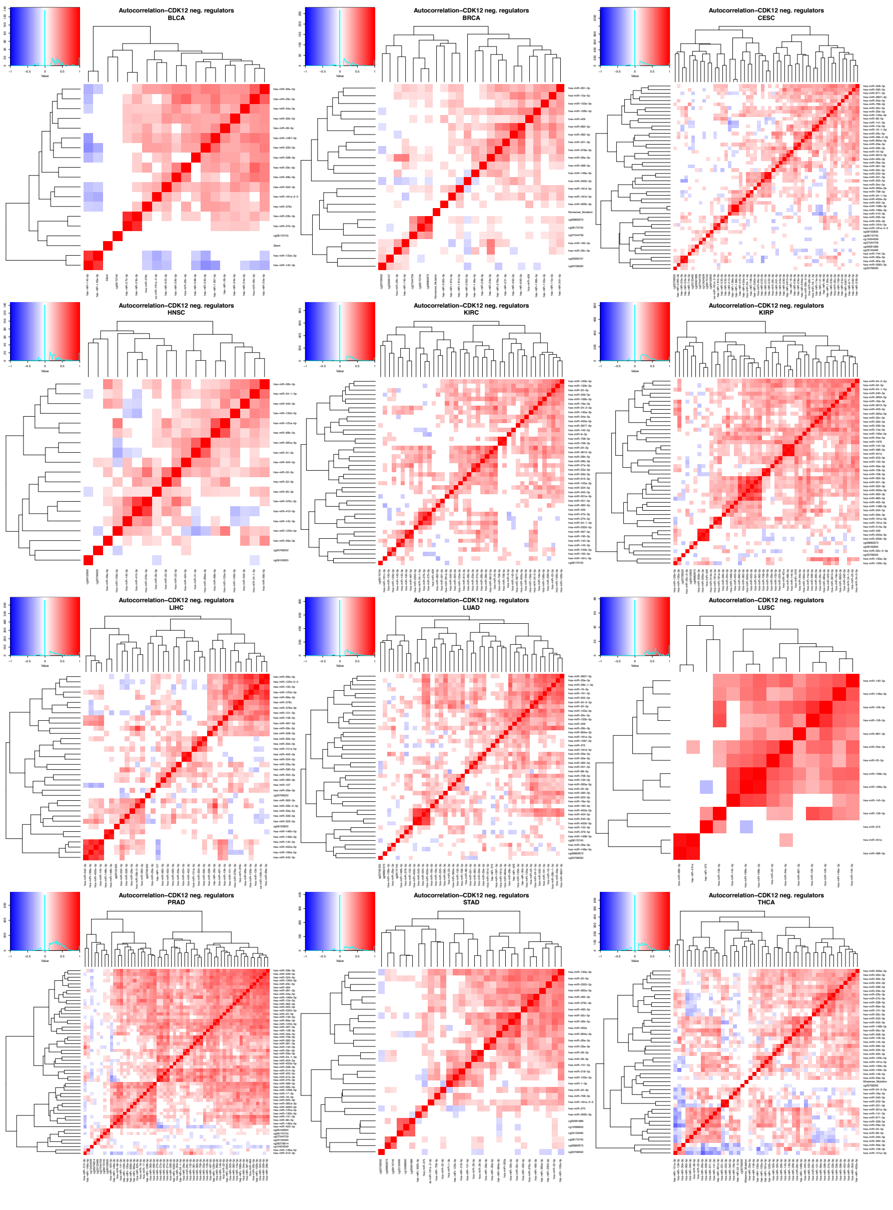

Supplementary Figure 21. Autocorrelation heatmap for the expression of the identified negative regulators of CDK12, across the 12 cancer types considered.

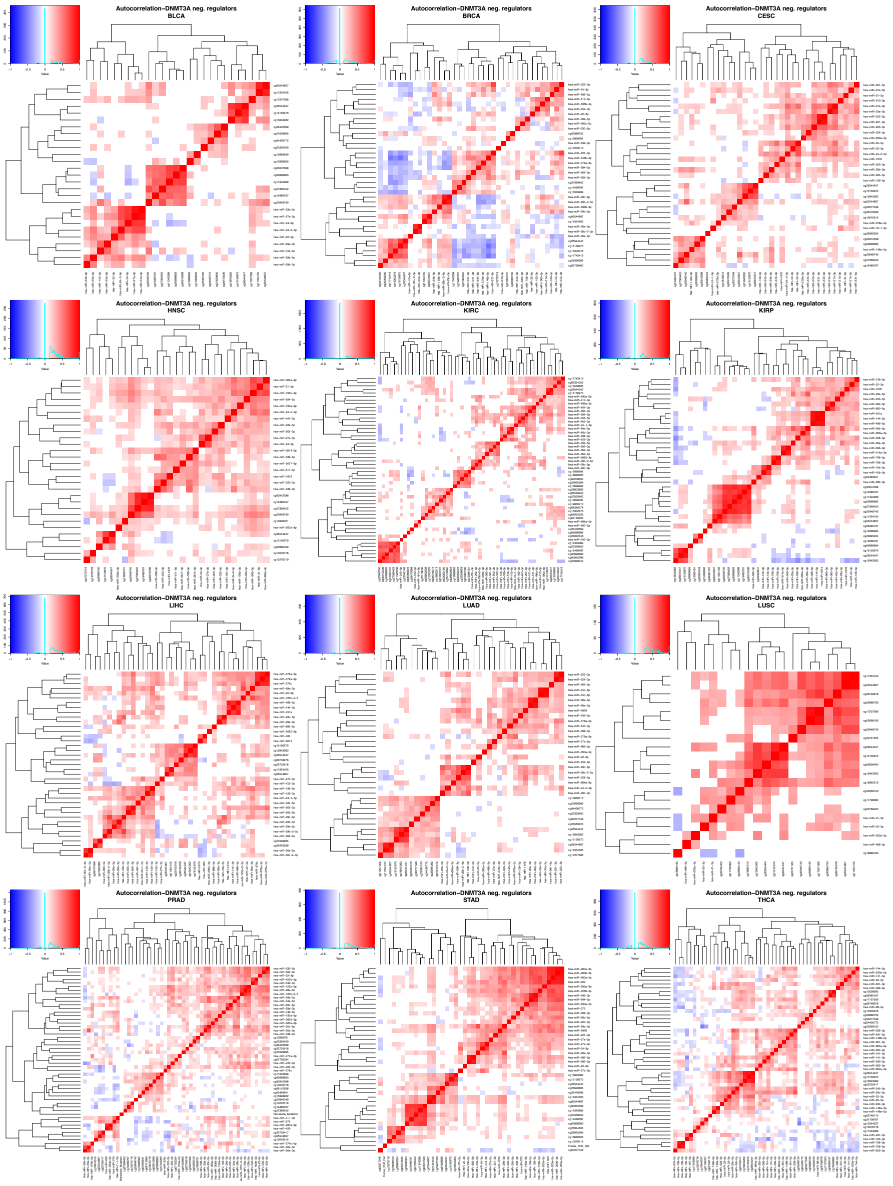

Supplementary Figure 22. Autocorrelation heatmap for the expression of the identified negative regulators of DNMT3A, across the 12 cancer types considered.

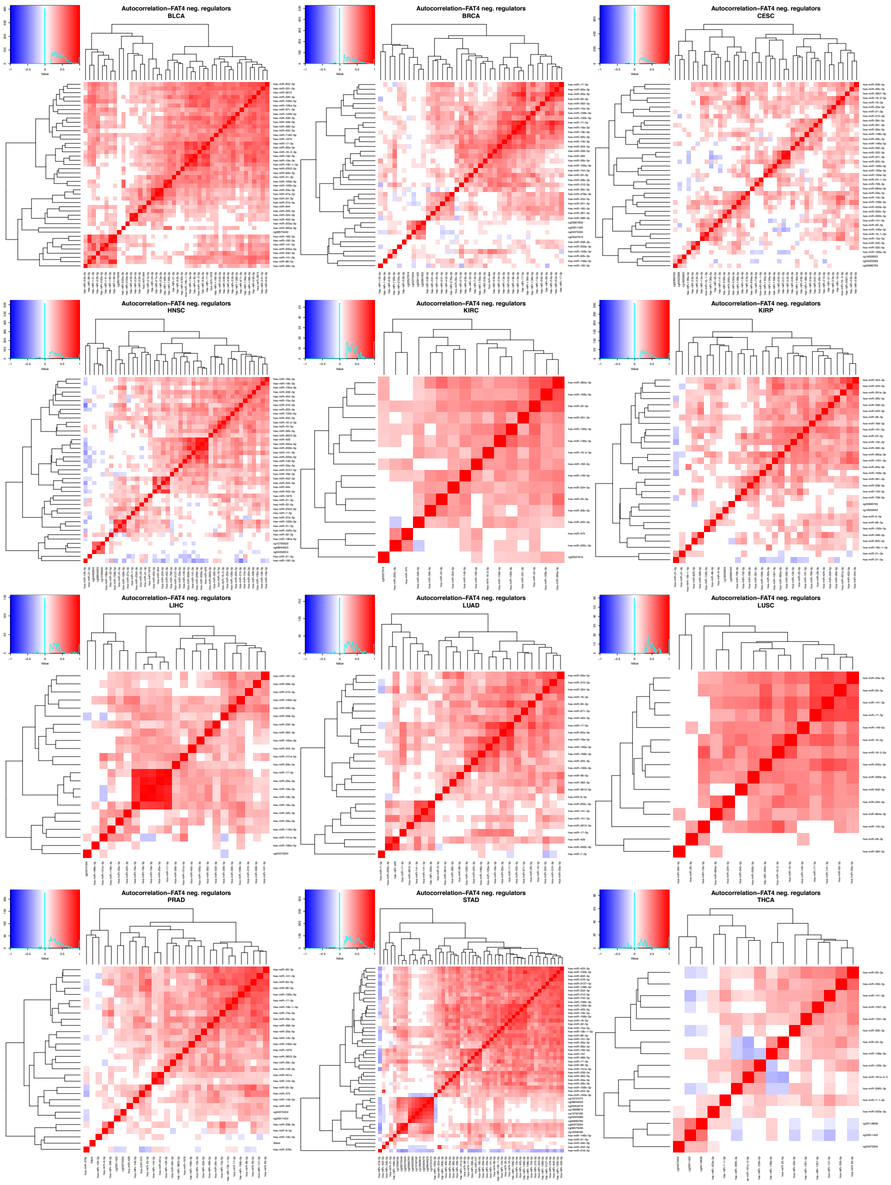

Supplementary Figure 23. Autocorrelation heatmap for the expression of the identified negative regulators of FAT4, across the 12 cancer types considered.

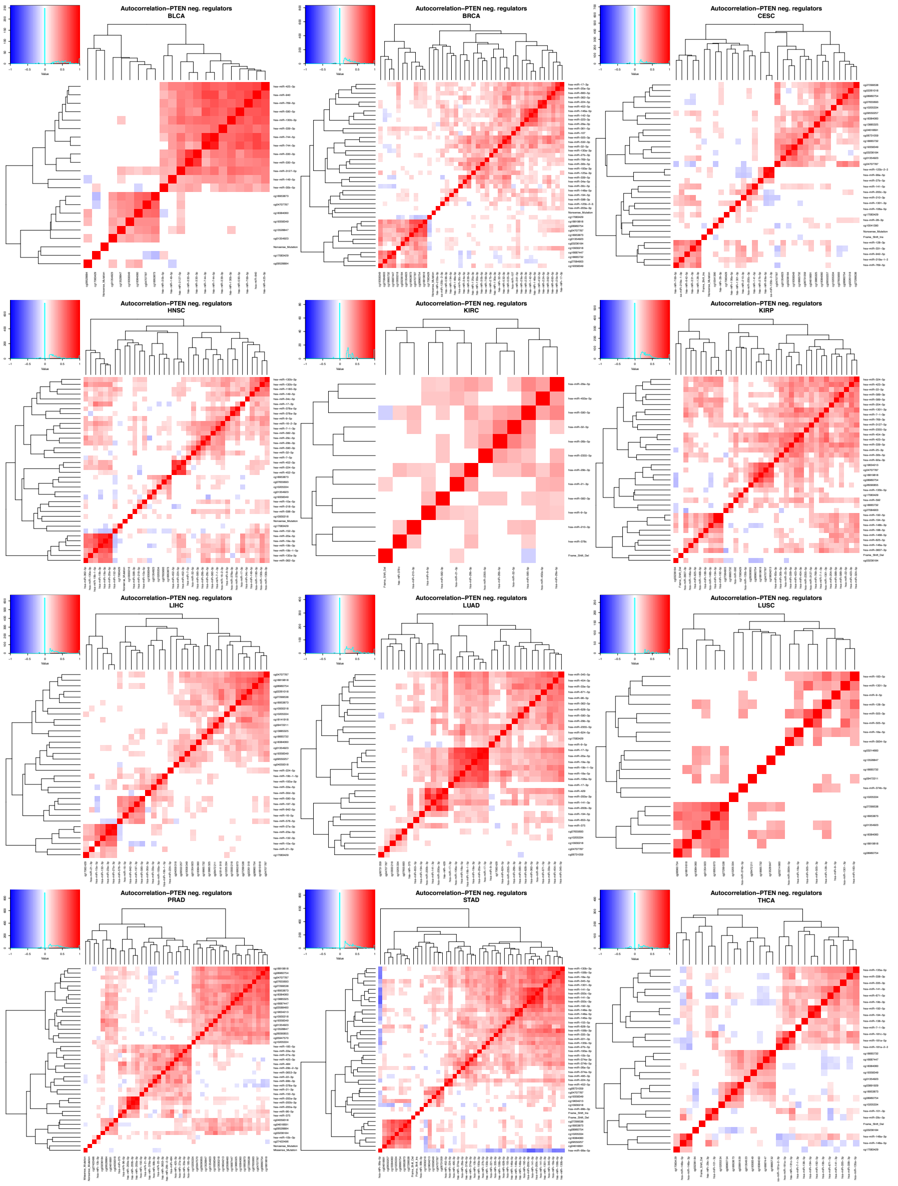

Supplementary Figure 24. Autocorrelation heatmap for the expression of the identified negative regulators of PTEN, across the 12 cancer types considered.

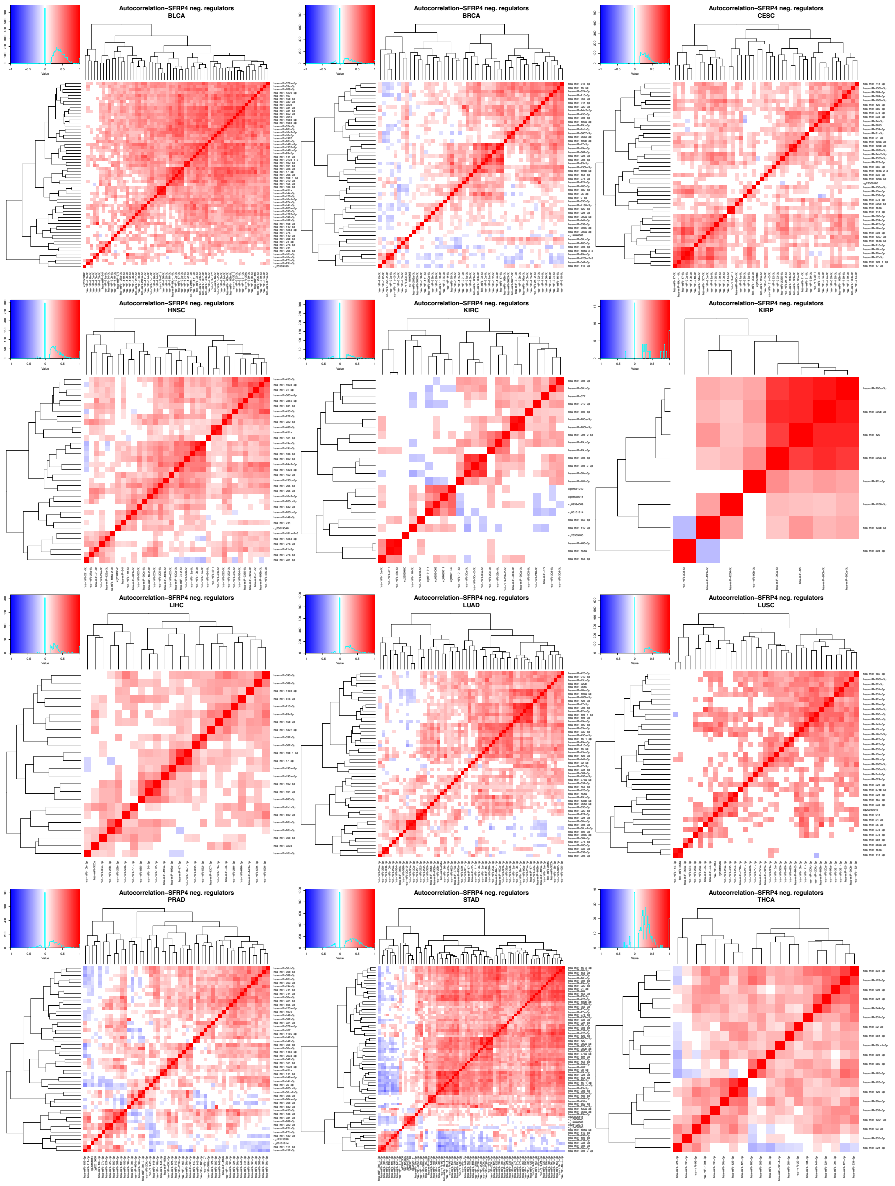

Supplementary Figure 25. Autocorrelation heatmap for the expression of the identified negative regulators of SFRP4, across the 12 cancer types considered.

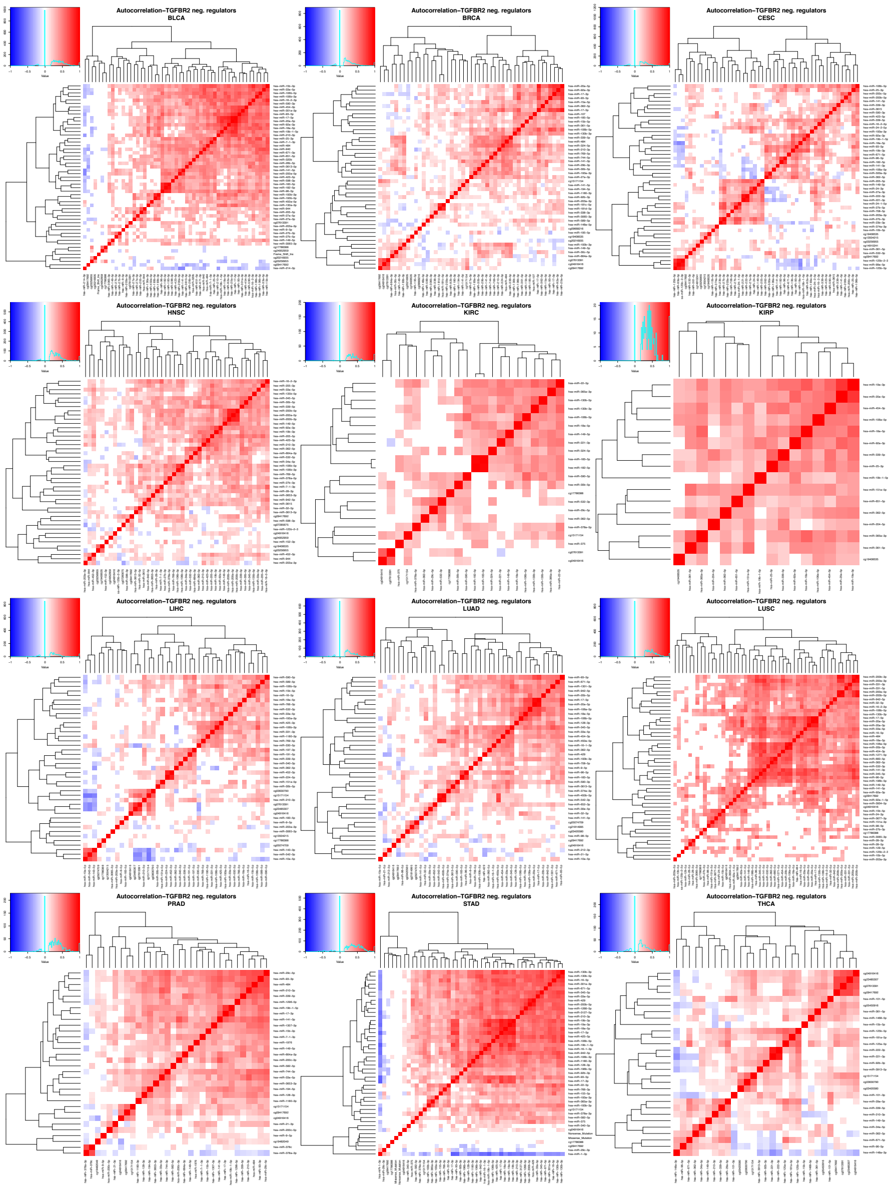

Supplementary Figure 26. Autocorrelation heatmap for the expression of the identified negative regulators of TGFBR2, across the 12 cancer types considered.

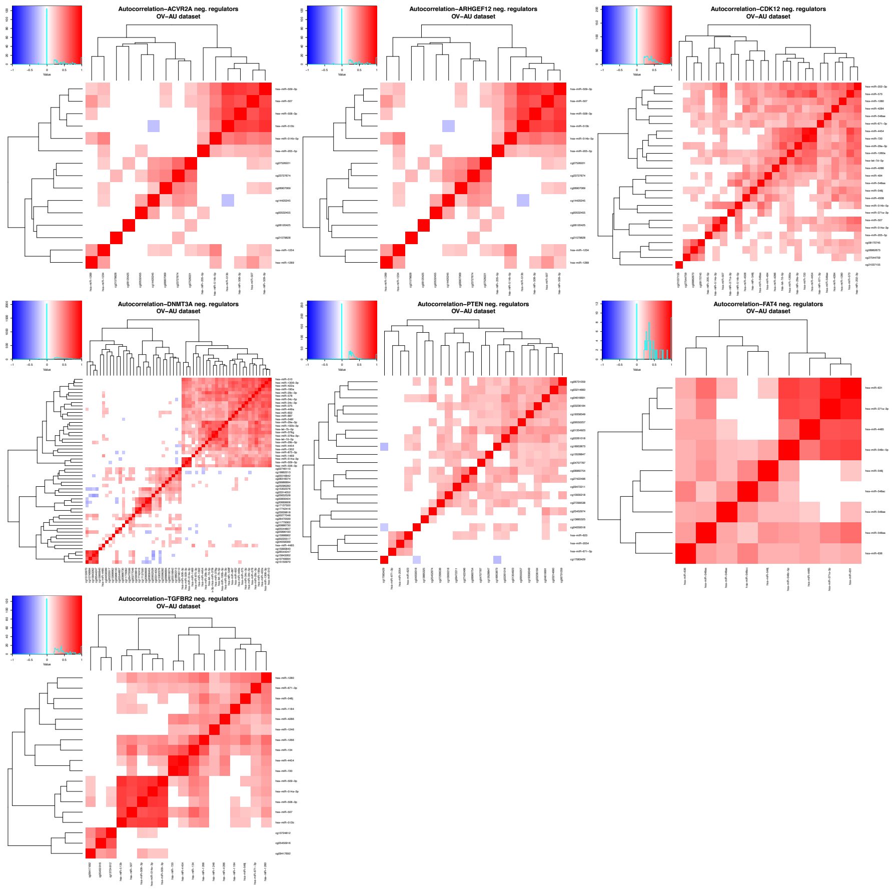

Supplementary Figure 27. Autocorrelation heatmap for the expression of the identified negative regulators of each of the 8 significant TSG identified in this study in an independent ovarian cancer dataset. DNMT3A ($\Pi_{\rho_{\text{miR-miR}}} - \Pi_{\rho_{\text{miR-meth}}} = 0.11$, $\Pi_{\rho_{\text{miR-miR}}} - \Pi_{\rho_{\text{meth-meth}}} = 0.01$, and $\Pi_{\rho_{\text{meth-meth}}} - \Pi_{\rho_{\text{miR-meth}}} = 0.99$) and PTEN ($\Pi_{\rho_{\text{miR-miR}}} - \Pi_{\rho_{\text{miR-meth}}} = 0.18$, $\Pi_{\rho_{\text{miR-miR}}} - \Pi_{\rho_{\text{meth-meth}}} = 0.05$, and $\Pi_{\rho_{\text{meth-meth}}} - \Pi_{\rho_{\text{miR-meth}}} = 1$) tend towards exclusivity in this dataset.

Supplementary Note 10. TSG expression and mutation status, miRNA expression, and methylation status

The following pages contain figures representing an analysis first showing the expression of each of the TSG grouped by regulatory type in Supplementary figures 28- 37. For each tumour suppressor gene considered, the miRNA associated to it were those identified as strong pan-cancer negative predictors of expression, and likewise the methylation probes associated to it were those identified as strong pan-cancer negative predictors of expression. Groups of miRNA high (+) and low (-) were defined by median expression of these TSG-associated miRNA above or below the median for a given set of samples of a particular tumour type. Methylation high (+) and low (-) groups were defined analogously. Mutation groups were defined by the presence (or absence) of non-silent mutations. Cases of TSG and tumour types for which, among the common samples displaying methylation, miRNA expression, mutation, and mRNA expression data, there were fewer than 5 samples with non-silent mutation were excluded from analysis.

Subsequently, in Supplementary figures 38- 46, we present the correlation of the log2 fold changes for the differentially expressed genes between samples stratified into two groups based on mutation status of the TSG, versus samples with no mutation, lowly methylated sites, and low miRNA expression. Results show that commonly differentially expressed genes among the two groups from the null case of no apparent regulation of the TSG, across cancer types for which at least 5 mutant cases were present, show strong correlation in log2 fold change, suggesting a similar transcriptomic phenotype afforded by methylation and/or miRNA regulation as mutation for these TSG. Differential expression of genes was computed by the EBSeq (Empiric Bayesian) approach to estimate differential expression of genes taken from RSEM non-normalised raw counts for mRNA expression data from the TCGA Firebrowse data portal. In all analyses an FDR of 0.05 was used.

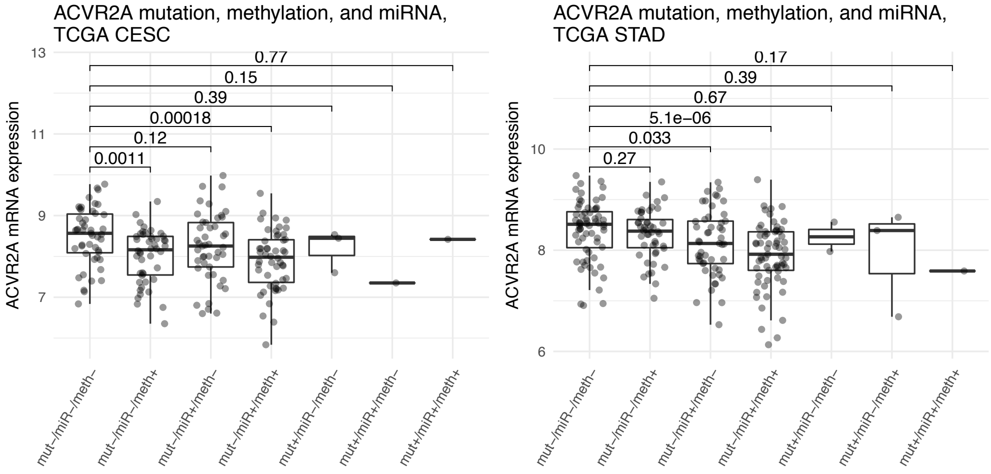

Supplementary Figure 28. Boxplots showing expression of ACVR2A across the distinct regulatory subgroups, across tumour types with at least 5 samples showing non-silent mutation. miRNA status is determined by whether median of identified negatively associated miRNA show expression above or below median value across samples, methylation status is defined analogously.

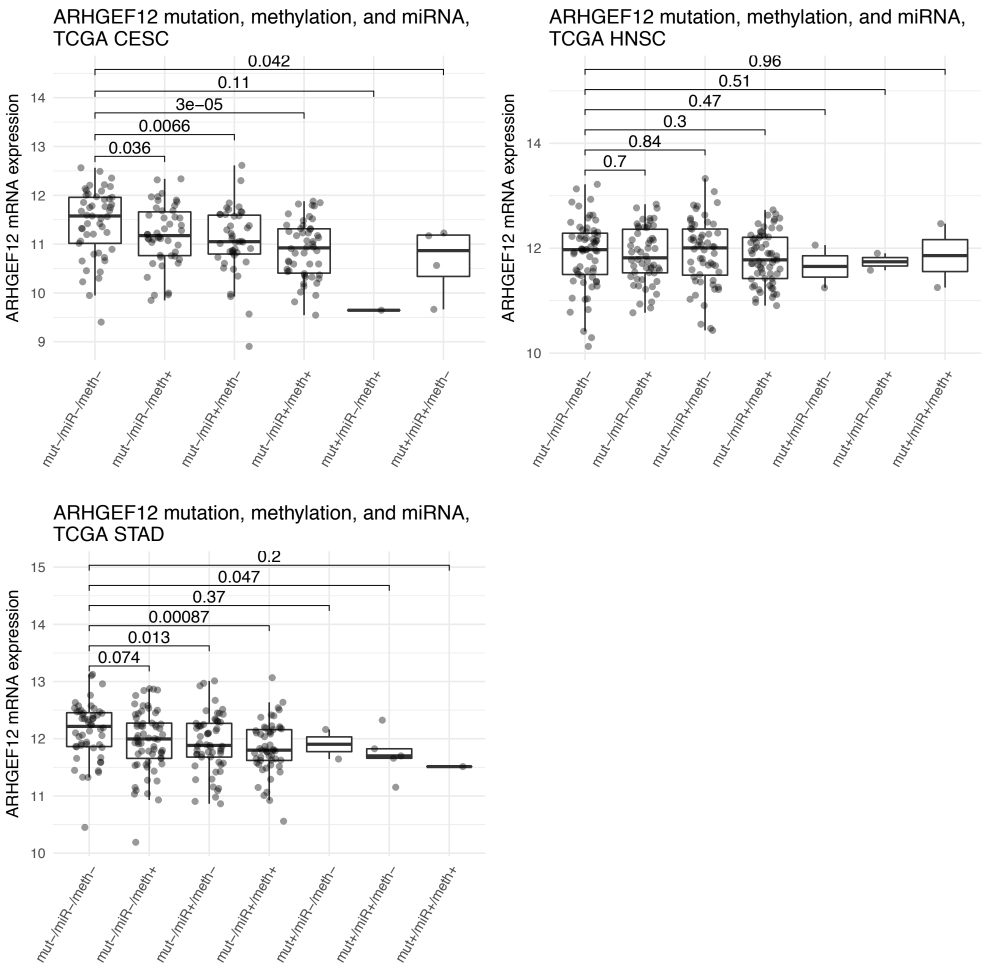

Supplementary Figure 29. Boxplots showing expression of ARHGEF12 across the distinct regulatory subgroups, across tumour types with at least 5 samples showing non-silent mutation. miRNA status is determined by whether median of identified negatively associated miRNA show expression above or below median value across samples, methylation status is defined analogously.

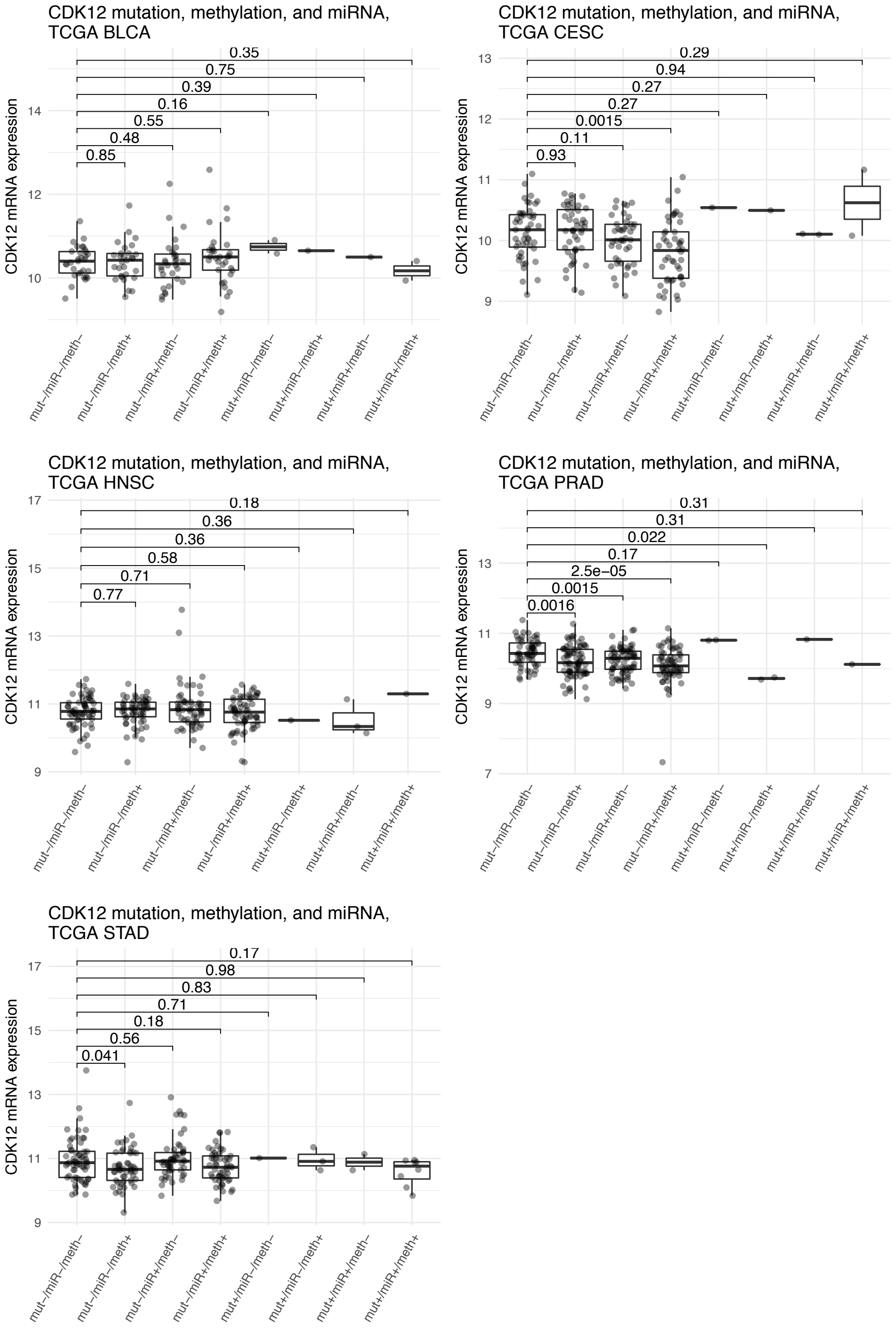

Supplementary Figure 30. Boxplots showing expression of CDK12 across the distinct regulatory subgroups, across tumour types with at least 5 samples showing non-silent mutation. miRNA status is determined by whether median of identified negatively associated miRNA show expression above or below median value across samples, methylation status is defined analogously.

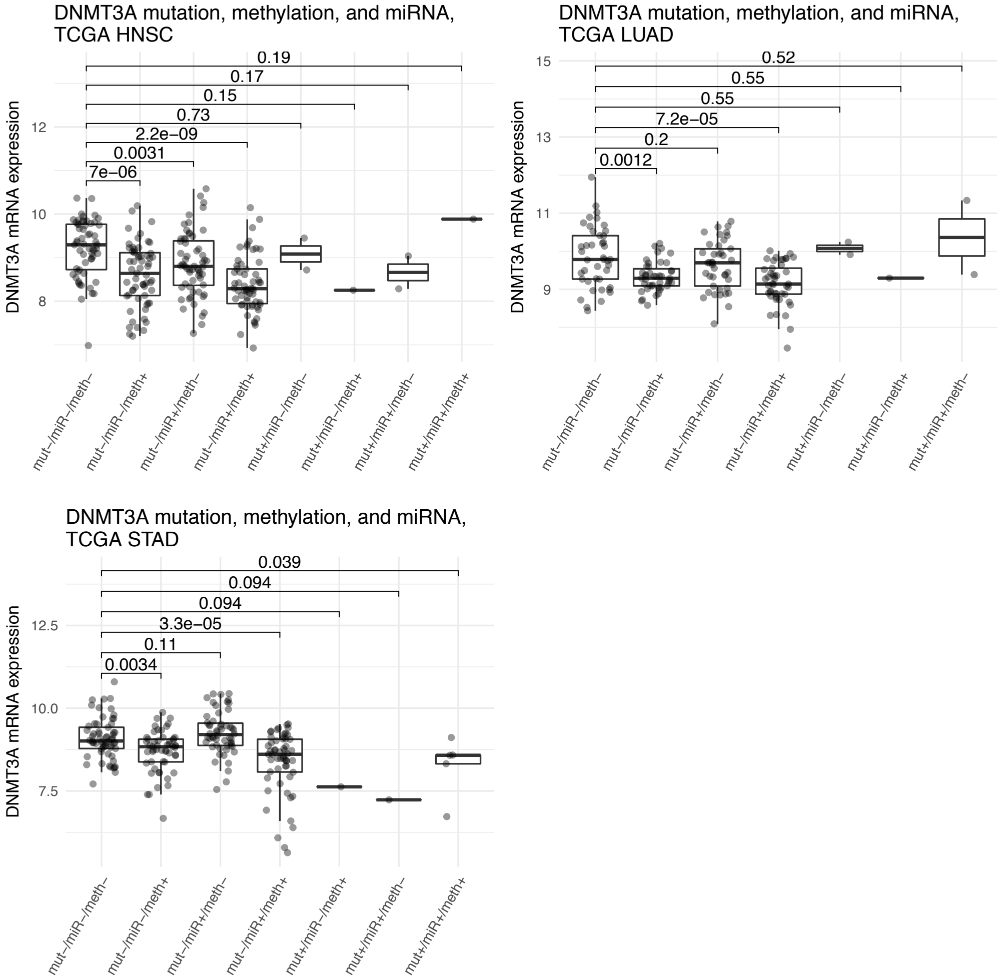

Supplementary Figure 31. Boxplots showing expression of DNMT3A across the distinct regulatory subgroups, across tumour types with at least 5 samples showing non-silent mutation. miRNA status is determined by whether median of identified negatively associated miRNA show expression above or below median value across samples, methylation status is defined analogously.

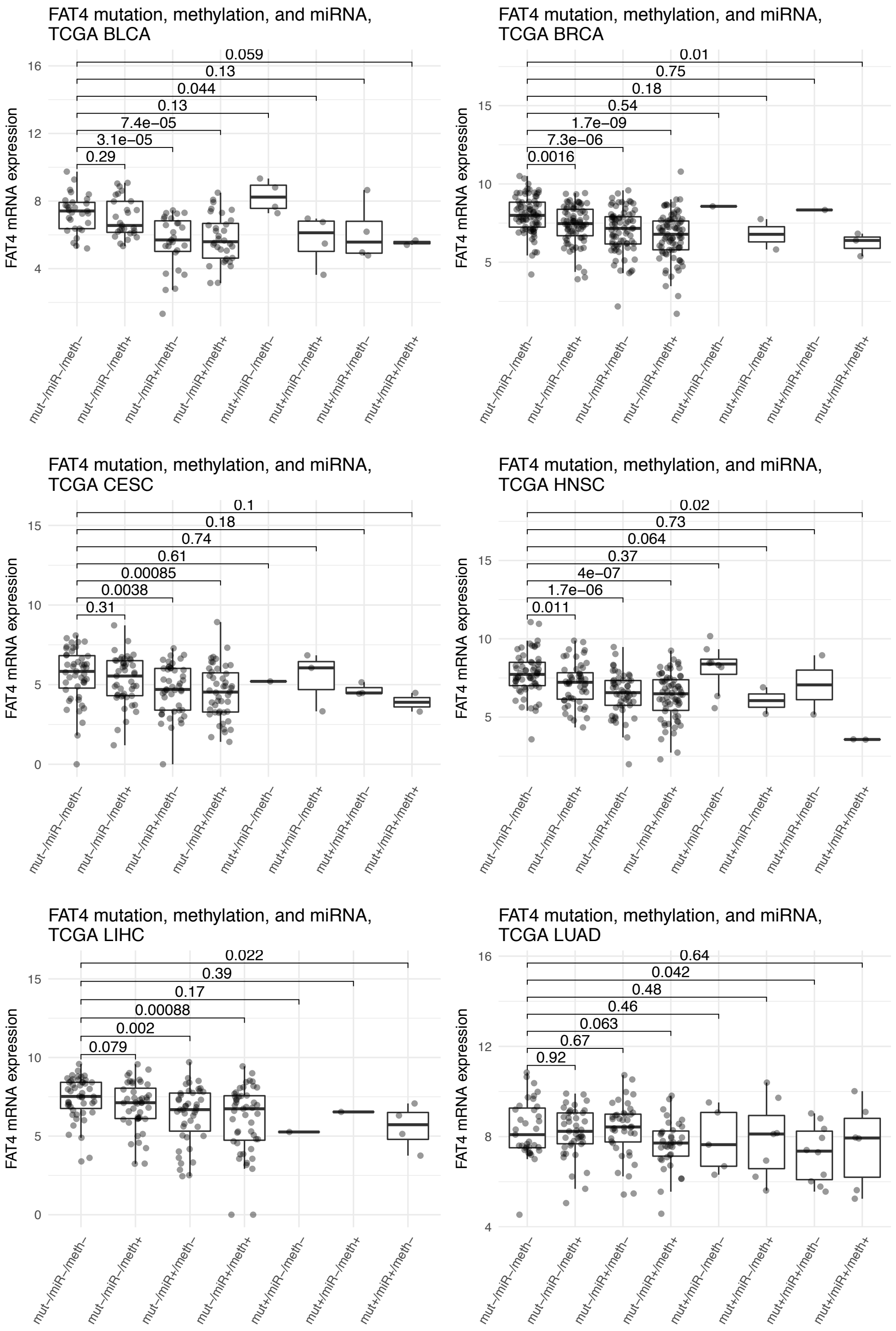

Supplementary Figure 32. Boxplots showing expression of FAT4 across the distinct regulatory subgroups, across tumour types with at least 5 samples showing non-silent mutation. miRNA status is determined by whether median of identified negatively associated miRNA show expression above or below median value across samples, methylation status is defined analogously.

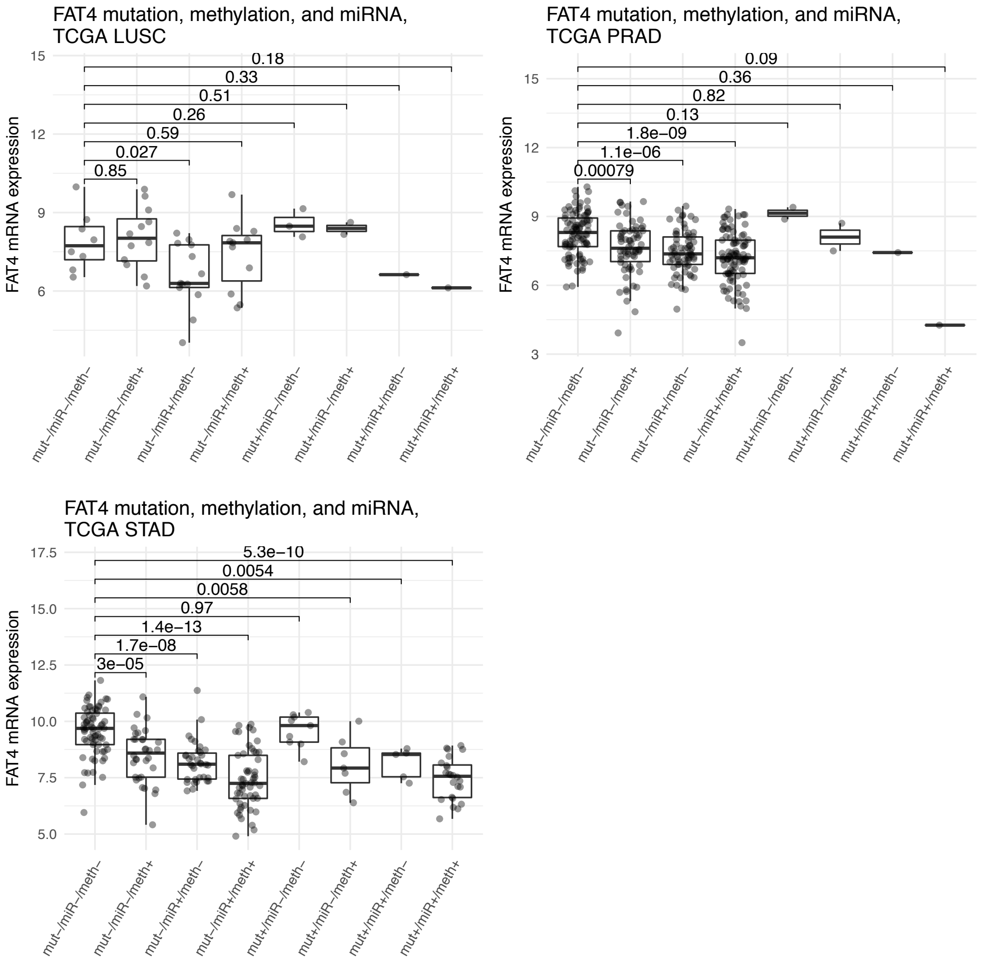

Supplementary Figure 33. Boxplots showing expression of FAT4 across the distinct regulatory subgroups, across tumour types with at least 5 samples showing non-silent mutation. miRNA status is determined by whether median of identified negatively associated miRNA show expression above or below median value across samples, methylation status is defined analogously.

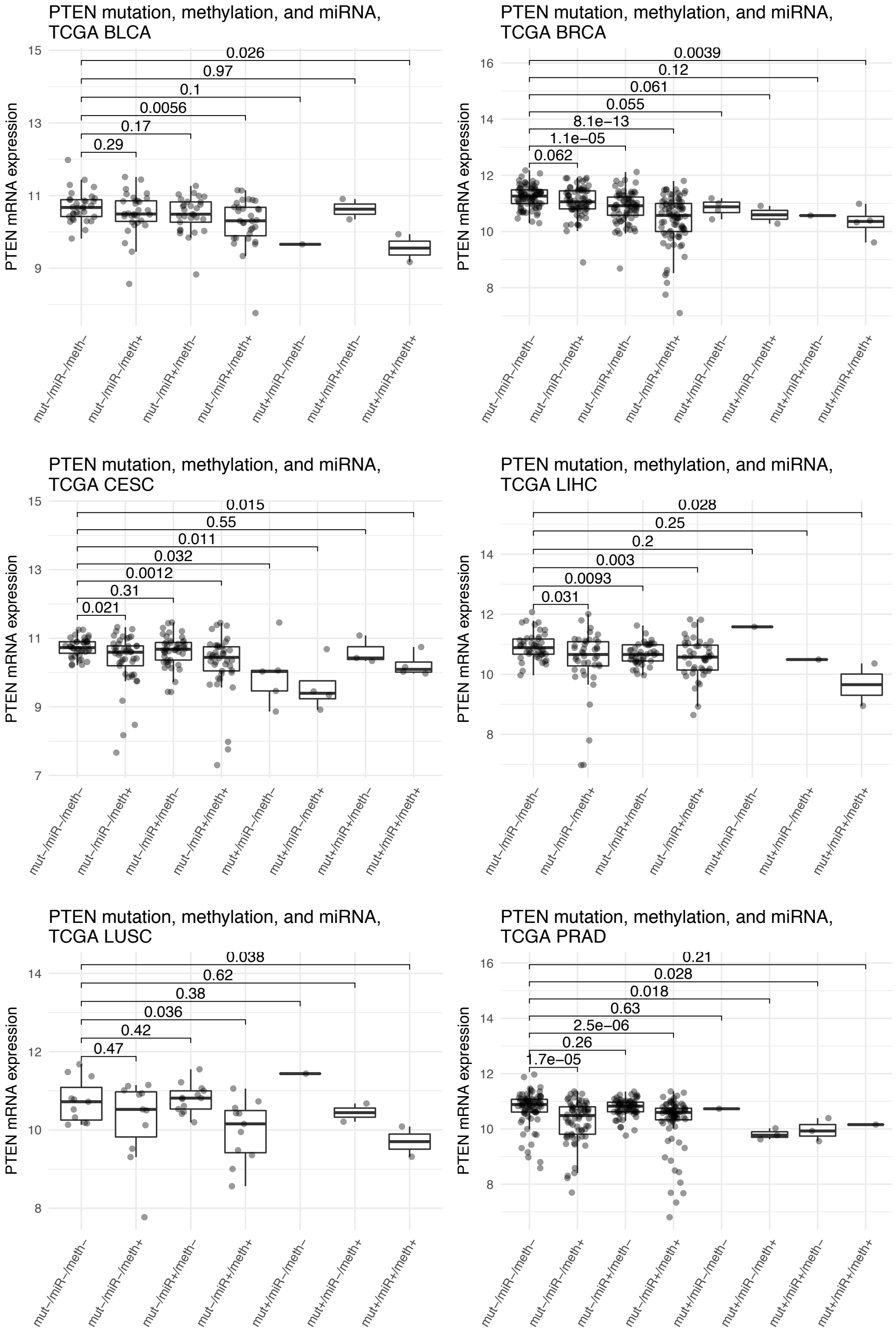

Supplementary Figure 34. Boxplots showing expression of PTEN across the distinct regulatory subgroups, across tumour types with at least 5 samples showing non-silent mutation. miRNA status is determined by whether median of identified negatively associated miRNA show expression above or below median value across samples, methylation status is defined analogously.

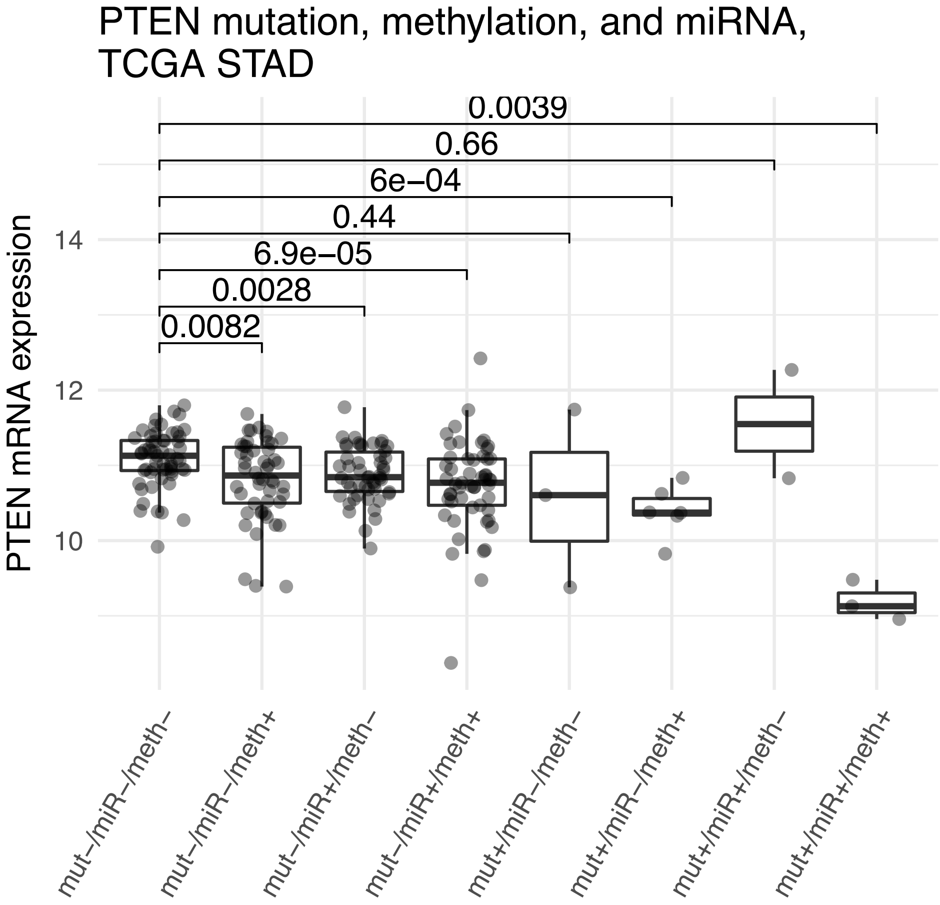

Supplementary Figure 35. Boxplots showing expression of PTEN across the distinct regulatory subgroups, across tumour types with at least 5 samples showing non-silent mutation. miRNA status is determined by whether median of identified negatively associated miRNA show expression above or below median value across samples, methylation status is defined analogously.

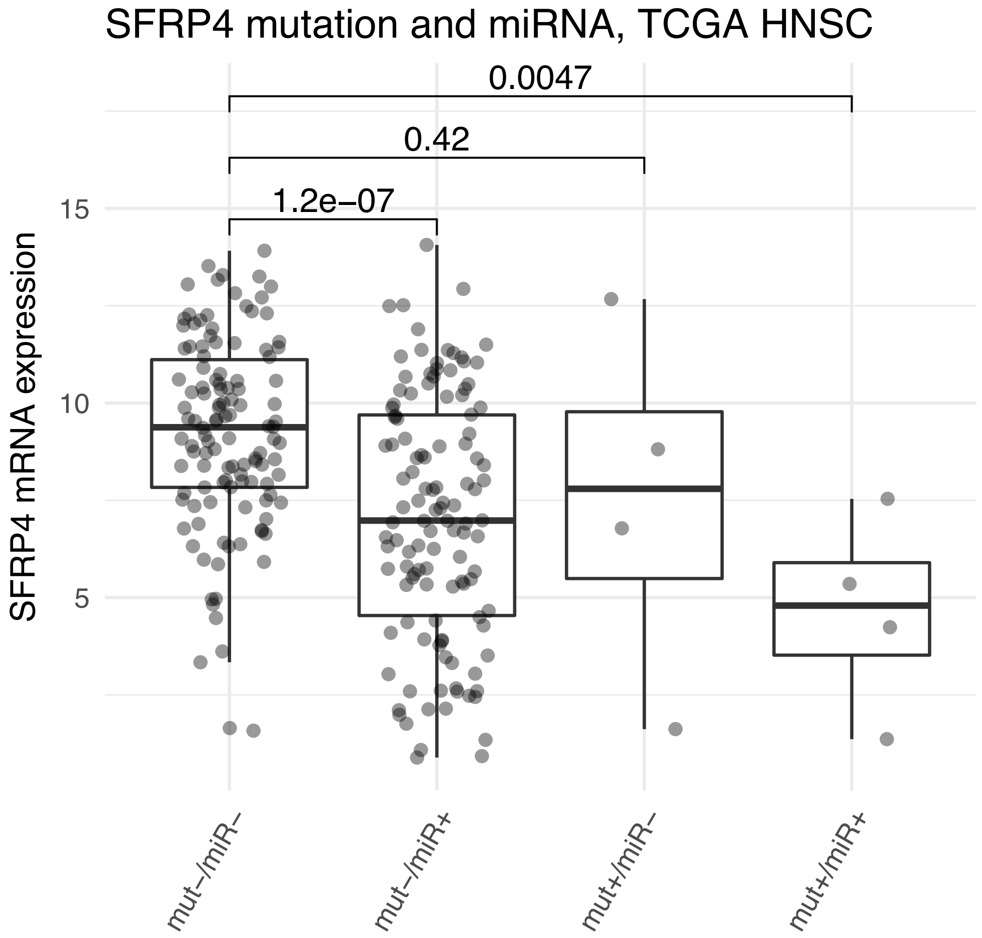

Supplementary Figure 36. Boxplots showing expression of SFRP4 across the distinct regulatory subgroups, across tumour types with at least 5 samples showing non-silent mutation. miRNA status is determined by whether median of identified negatively associated miRNA show expression above or below median value across samples.

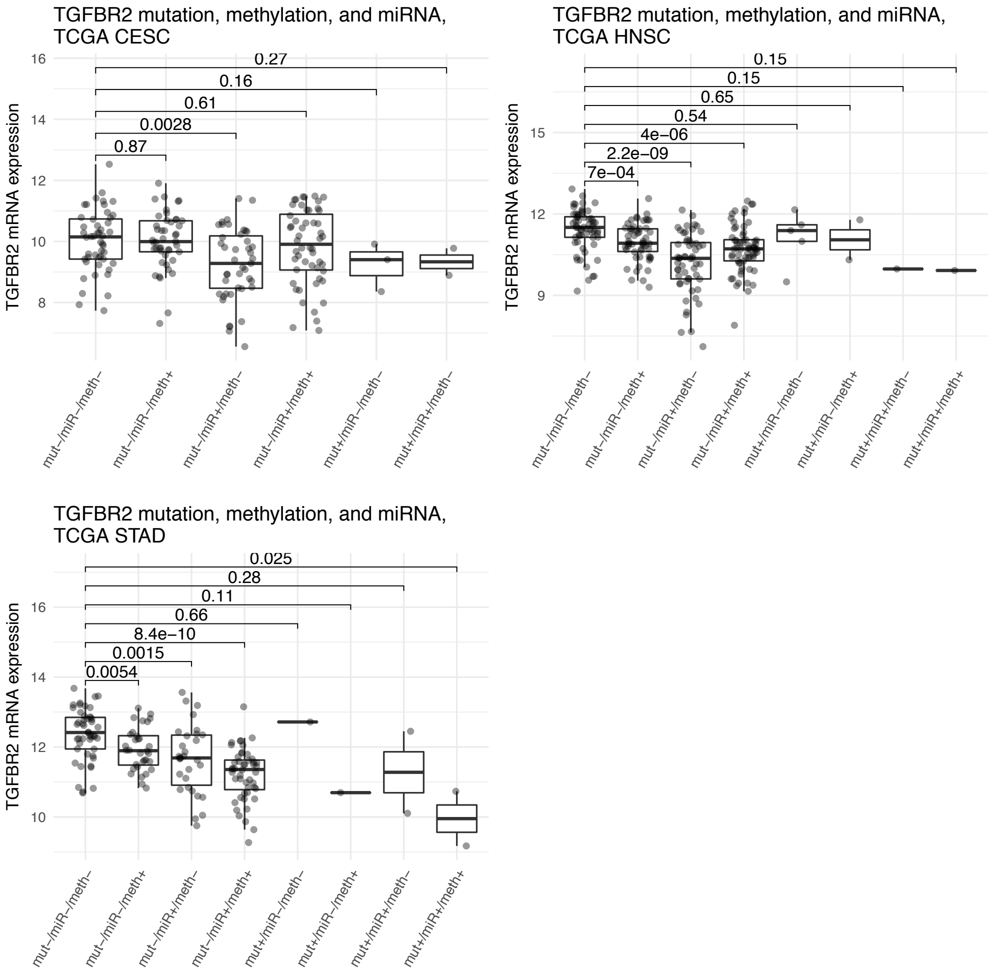

Supplementary Figure 37. Boxplots showing expression of TGFBR2 across the distinct regulatory subgroups, across tumour types with at least 5 samples showing non-silent mutation. miRNA status is determined by whether median of identified negatively associated miRNA show expression above or below median value across samples, methylation status is defined analogously.

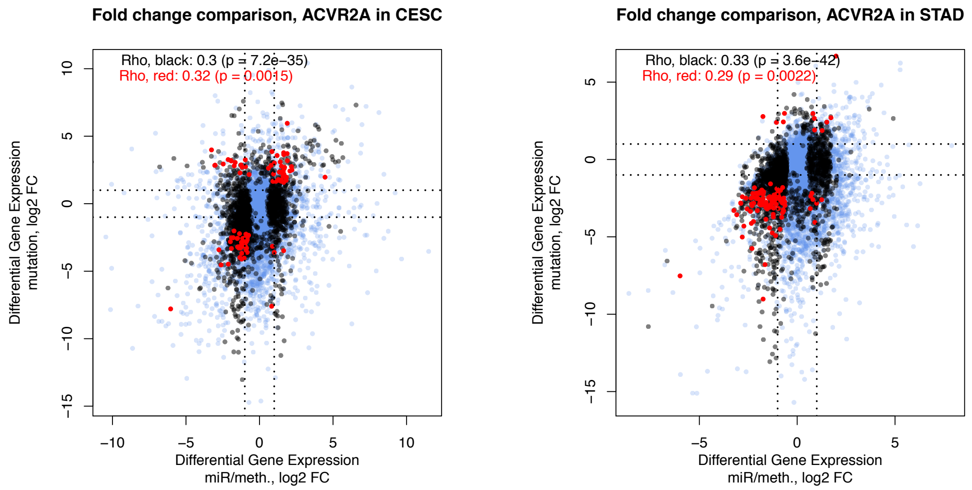

Supplementary Figure 38. Fold change (log2 transformed) for differentially expressed genes in ACVR2A mutated cases versus non-mutated, miRNA low, methylation-low samples was plotted against fold change (log2 transformed) for differentially expressed genes in unmutated ACVR2A miRNA high and/or methylation high versus non-mutated, miRNA low, methylation-low samples. Genes in black are differentially expressed in one of the two groups, genes in red are commonly differentially expressed, and genes represented by blue points are not differentially expressed in either case. Spearman's rho is computed both for black points and red points as given in plots.

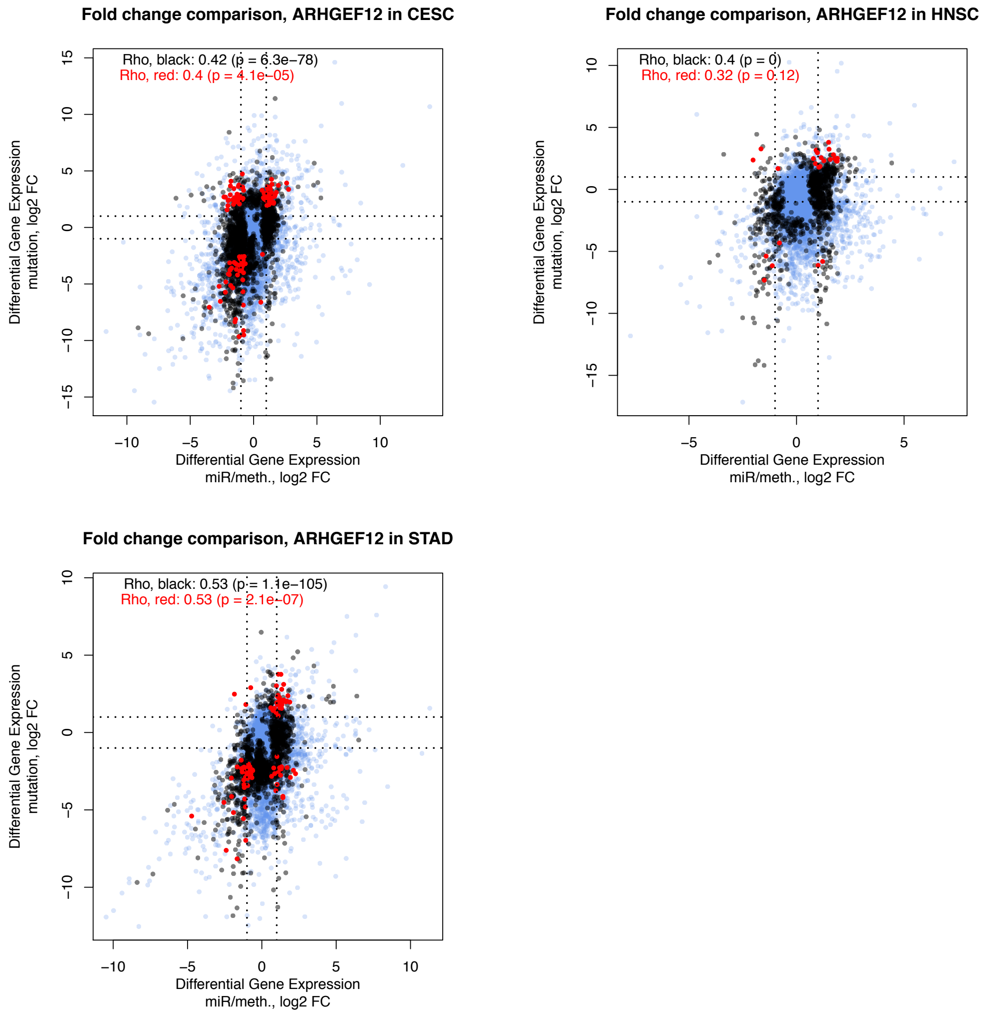

Supplementary Figure 39. Fold change (log2 transformed) for differentially expressed genes in ARHGEF12 mutated cases versus non-mutated, miRNA low, methylation-low samples was plotted against fold change (log2 transformed) for differentially expressed genes in unmutated ARHGEF12 miRNA high and/or methylation high versus non-mutated, miRNA low, methylation-low samples. Genes in black are differentially expressed in one of the two groups, genes in red are commonly differentially expressed, and genes represented by blue points are not differentially expressed in either case. Spearman's rho is computed both for black points and red points as given in plots.

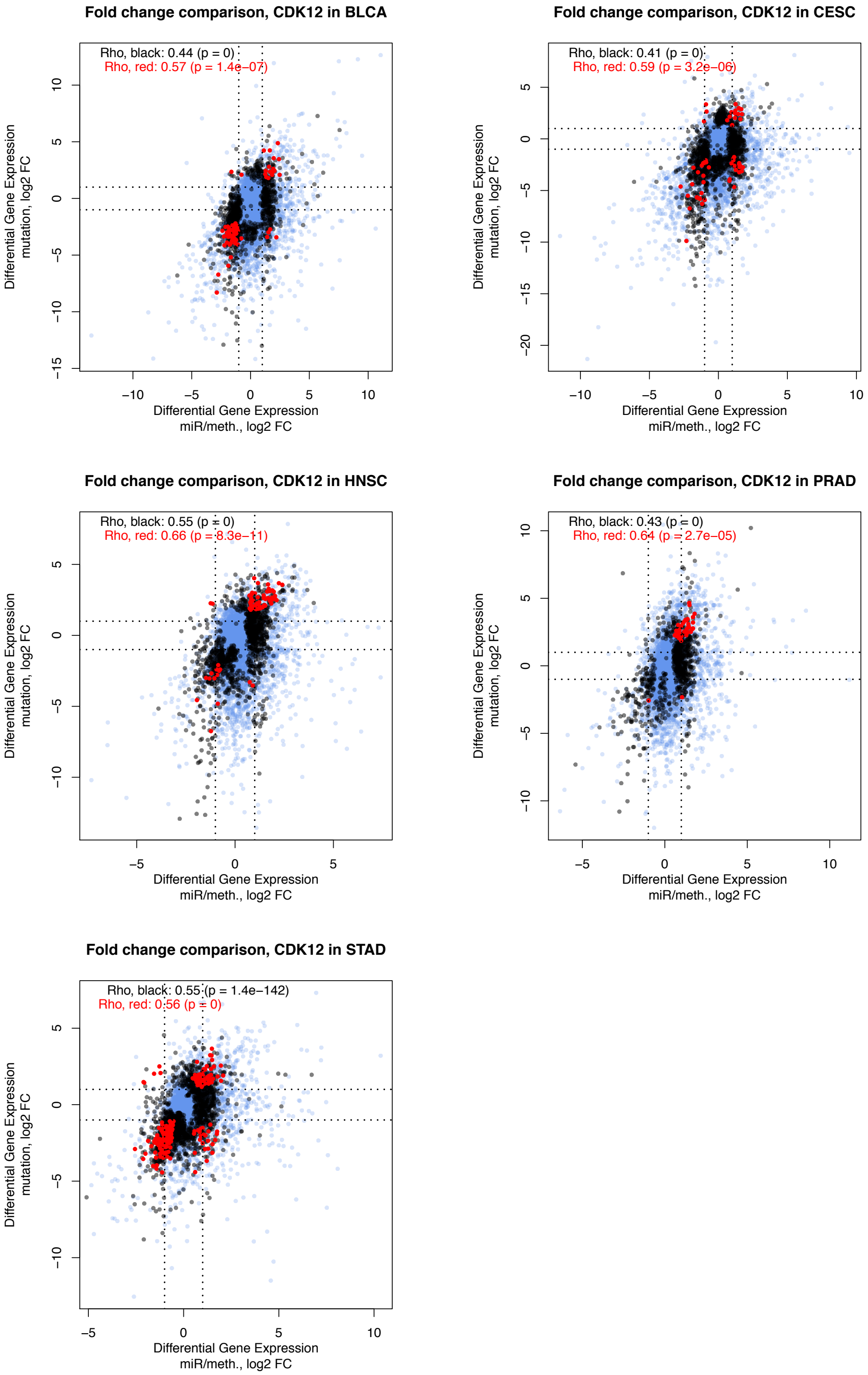

Supplementary Figure 40. Fold change (log2 transformed) for differentially expressed genes in CDK12 mutated cases versus non-mutated, miRNA low, methylation-low samples was plotted against fold change (log2 transformed) for differentially expressed genes in unmutated CDK12 miRNA high and/or methylation high versus non-mutated, miRNA low, methylation-low samples. Genes in black are differentially expressed in one of the two groups, genes in red are commonly differentially expressed, and genes represented by blue points are not differentially expressed in either case. Spearman's rho is computed both for black points and red points as given in plots.

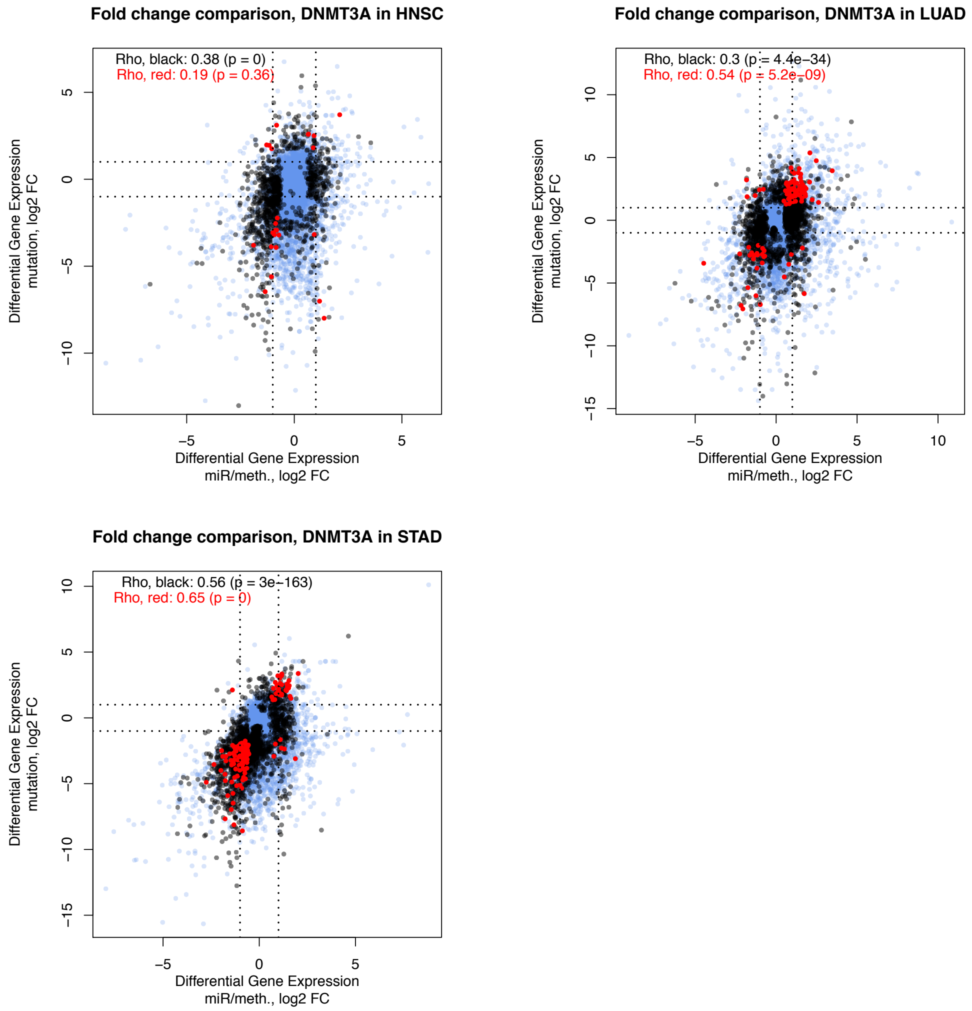

Supplementary Figure 41. Fold change (log2 transformed) for differentially expressed genes in DNMT3A mutated cases versus non-mutated, miRNA low, methylation-low samples was plotted against fold change (log2 transformed) for differentially expressed genes in unmutated DNMT3A miRNA high and/or methylation high versus non-mutated, miRNA low, methylation-low samples. Genes in black are differentially expressed in one of the two groups, genes in red are commonly differentially expressed, and genes represented by blue points are not differentially expressed in either case. Spearman's rho is computed both for black points and red points as given in plots.

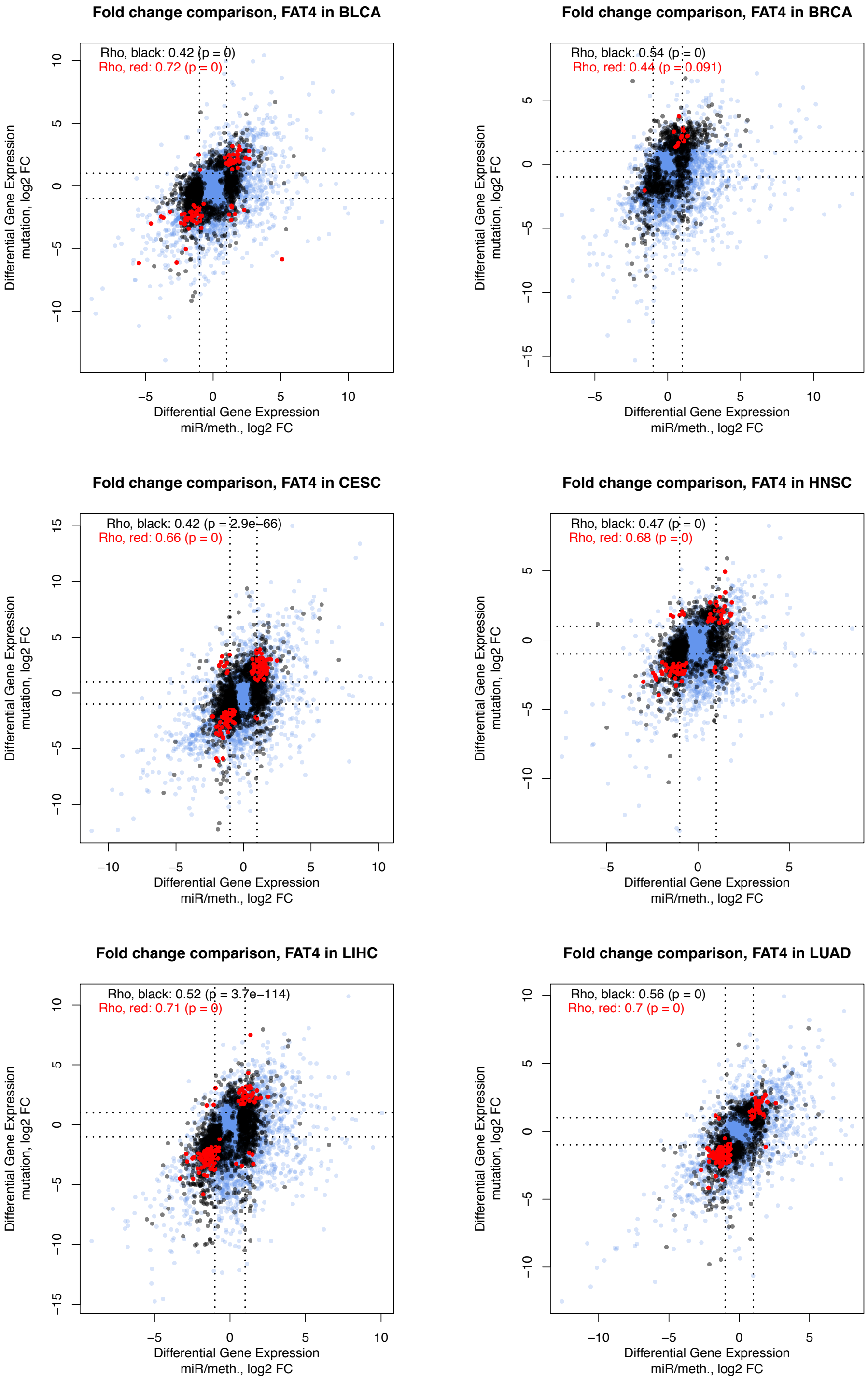

Supplementary Figure 42. Fold change (log2 transformed) for differentially expressed genes in FAT4 mutated cases versus non-mutated, miRNA low, methylation-low samples was plotted against fold change (log2 transformed) for differentially expressed genes in unmutated FAT4 miRNA high and/or methylation high versus non-mutated, miRNA low, methylation-low samples. Genes in black are differentially expressed in one of the two groups, genes in red are commonly differentially expressed, and genes represented by blue points are not differentially expressed in either case. Spearman's rho is computed both for black points and red points as given in plots.

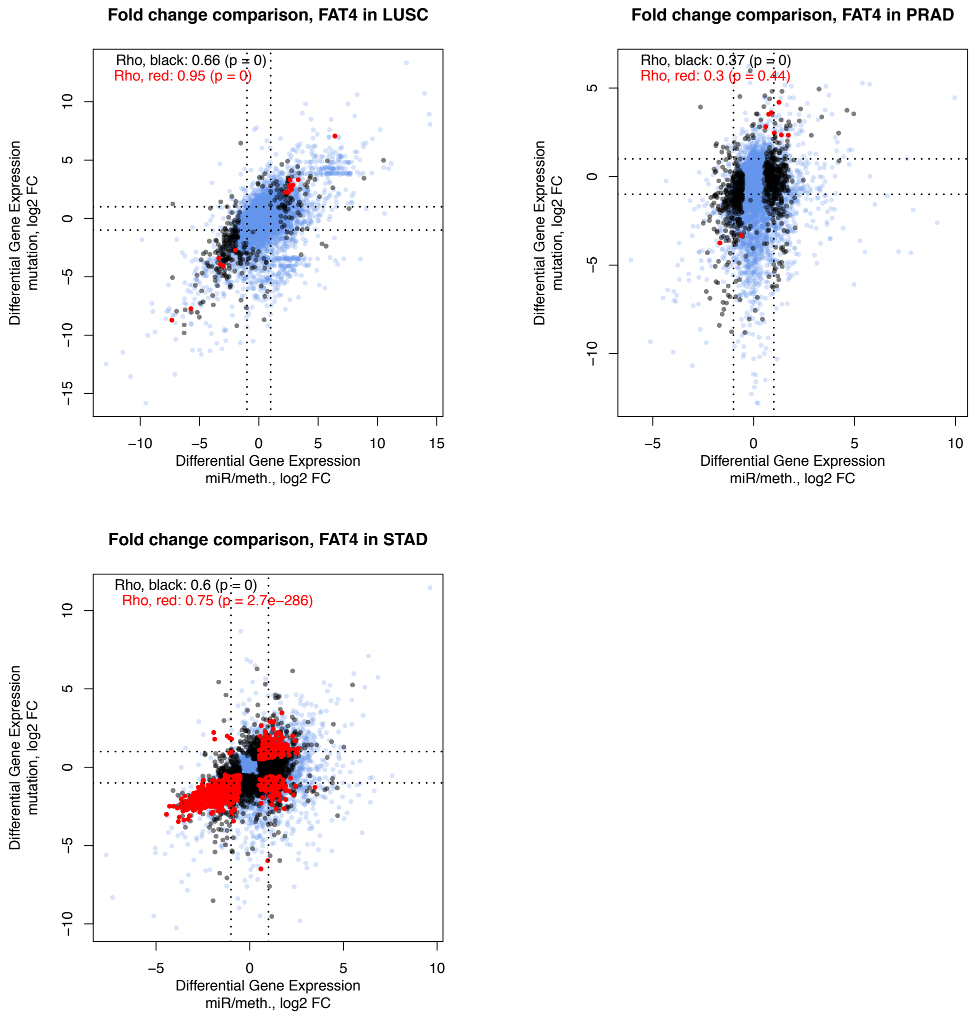

Supplementary Figure 43. Fold change (log2 transformed) for differentially expressed genes in FAT4 mutated cases versus non-mutated, miRNA low, methylation-low samples was plotted against fold change (log2 transformed) for differentially expressed genes in unmutated FAT4 miRNA high and/or methylation high versus non-mutated, miRNA low, methylation-low samples. Genes in black are differentially expressed in one of the two groups, genes in red are commonly differentially expressed, and genes represented by blue points are not differentially expressed in either case. Spearman's rho is computed both for black points and red points as given in plots.

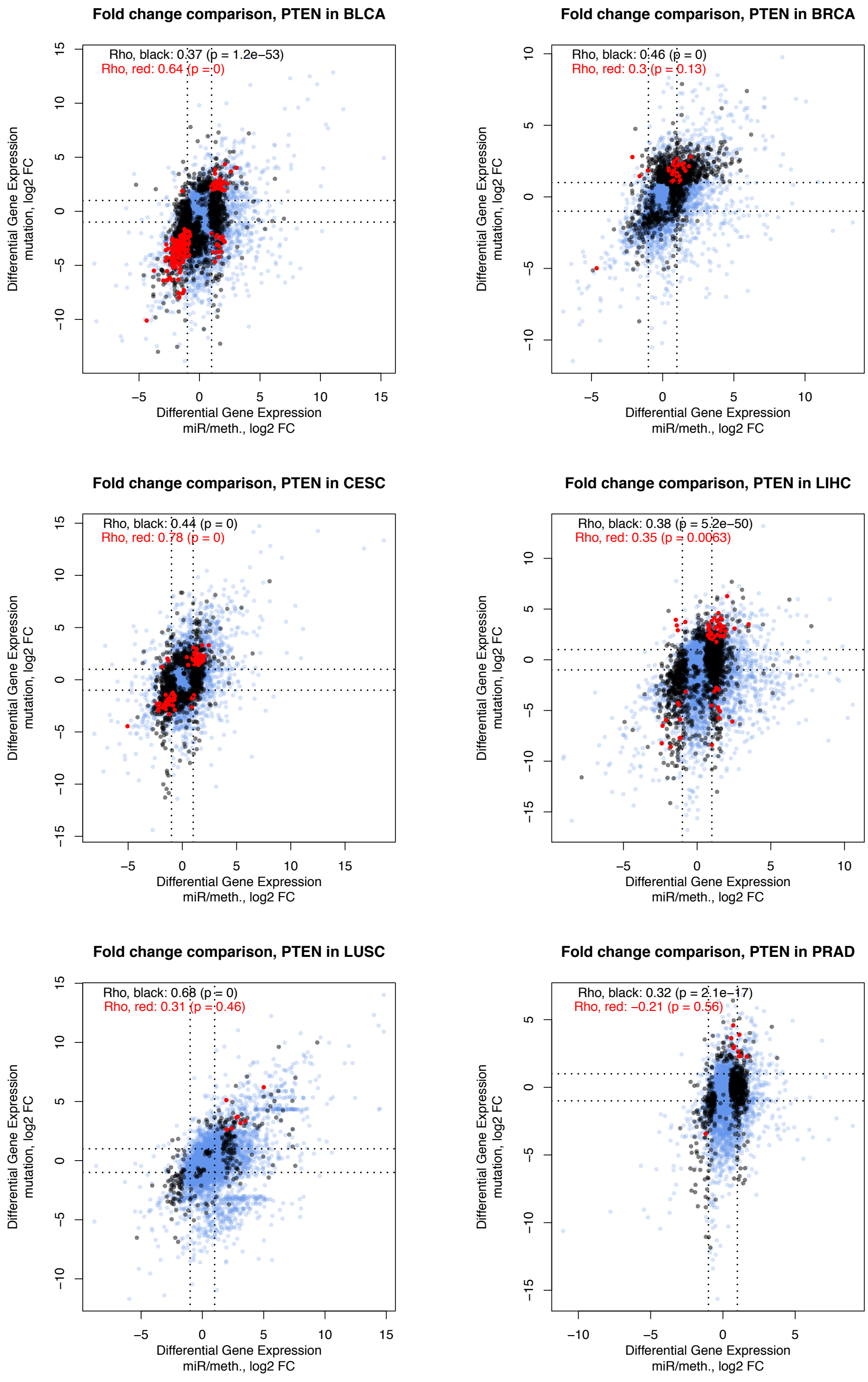

Supplementary Figure 44. Fold change (log2 transformed) for differentially expressed genes in PTEN mutated cases versus non-mutated, miRNA low, methylation-low samples was plotted against fold change (log2 transformed) for differentially expressed genes in unmutated PTEN miRNA high and/or methylation high versus non-mutated, miRNA low, methylation-low samples. Genes in black are differentially expressed in one of the two groups, genes in red are commonly differentially expressed, and genes represented by blue points are not differentially expressed in either case. Spearman's rho is computed both for black points and red points as given in plots.

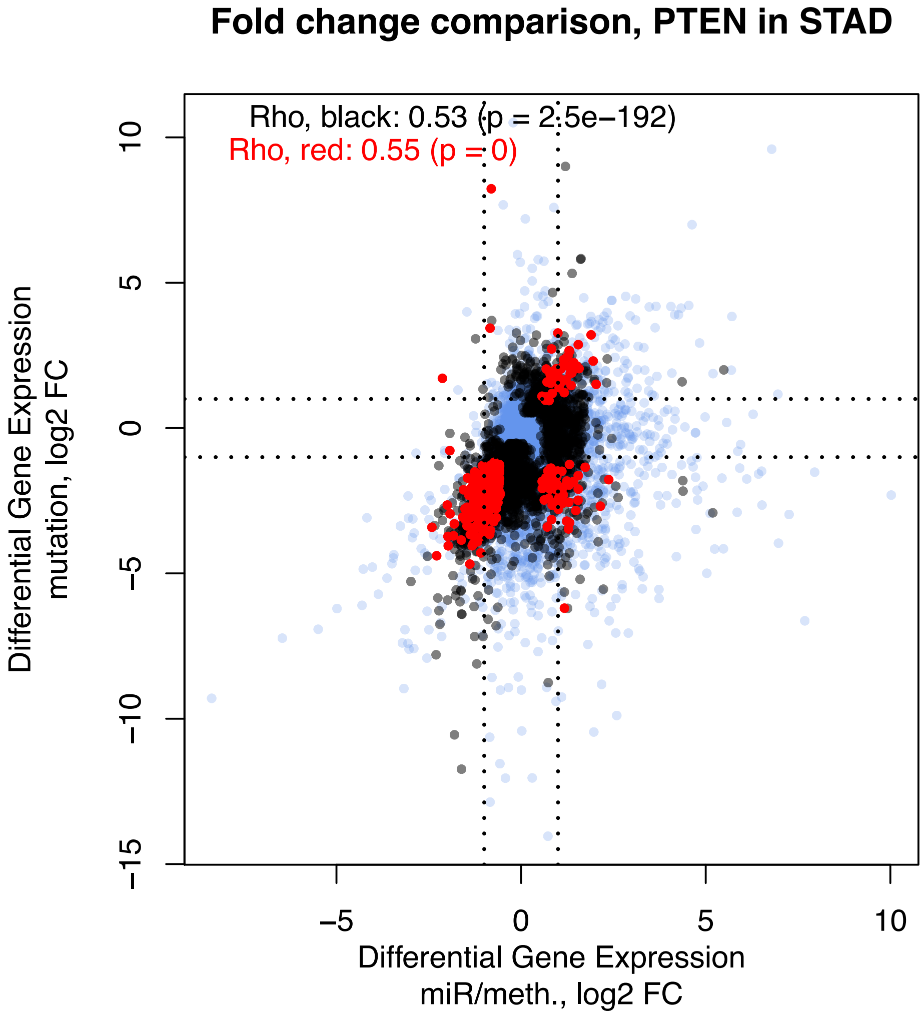

Supplementary Figure 45. Fold change (log2 transformed) for differentially expressed genes in PTEN mutated cases versus non-mutated, miRNA low, methylation-low samples was plotted against fold change (log2 transformed) for differentially expressed genes in unmutated PTEN miRNA high and/or methylation high versus non-mutated, miRNA low, methylation-low samples. Genes in black are differentially expressed in one of the two groups, genes in red are commonly differentially expressed, and genes represented by blue points are not differentially expressed in either case. Spearman's rho is computed both for black points and red points as given in plots.

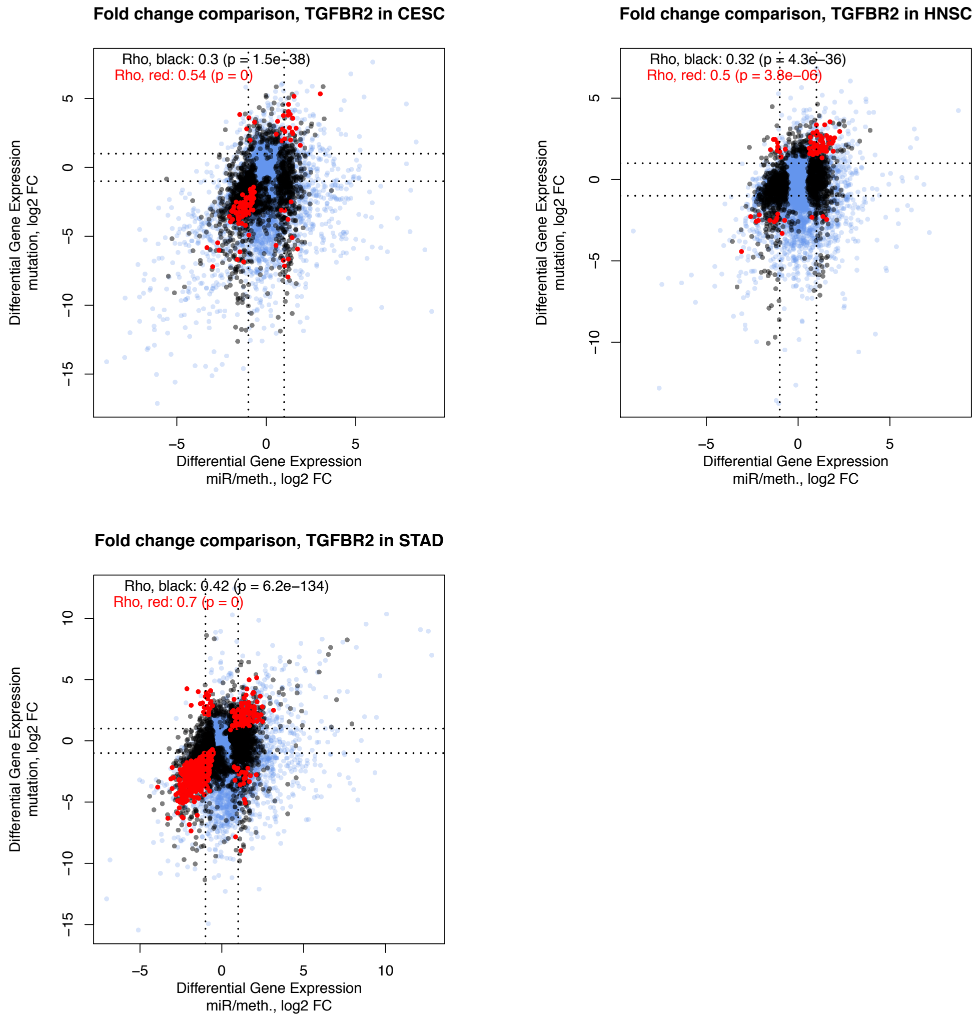

Supplementary Figure 46. Fold change (log2 transformed) for differentially expressed genes in TGFBR2 mutated cases versus non-mutated, miRNA low, methylation-low samples was plotted against fold change (log2 transformed) for differentially expressed genes in unmutated TGFBR2 miRNA high and/or methylation high versus non-mutated, miRNA low, methylation-low samples. Genes in black are differentially expressed in one of the two groups, genes in red are commonly differentially expressed, and genes represented by blue points are not differentially expressed in either case. Spearman's rho is computed both for black points and red points as given in plots.

Supplementary Note 11. ARHGEF12, SFRP4, and TGFBR2 association with breast cancer subtype

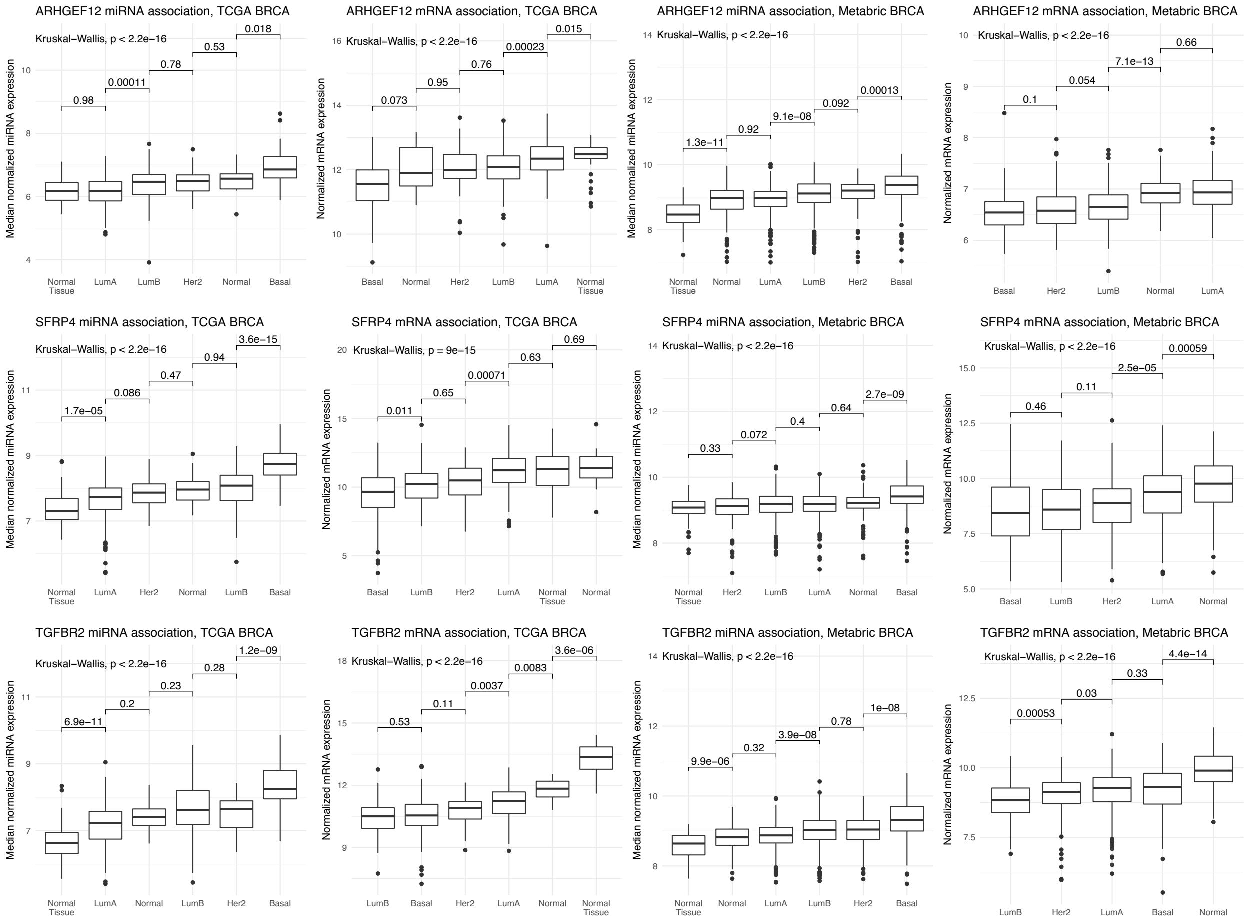

Supplementary Figure 47. Panels displaying the median normalised miRNA expression and normalised mRNA expression level associations of tumour suppressor genes and breast cancer molecular subtypes. Rows, descending, represent plots for ARHGEF12, SFRP4, and TGFBR2 respectively. The left two columns show median miRNA and mRNA expression for the TCGA breast cancer dataset, respectively. The right two columns show median miRNA and mRNA expression for the Metabric dataset, respectively.

Supplementary Note 12. MYC amplification status and TSG-associated miRNA expression

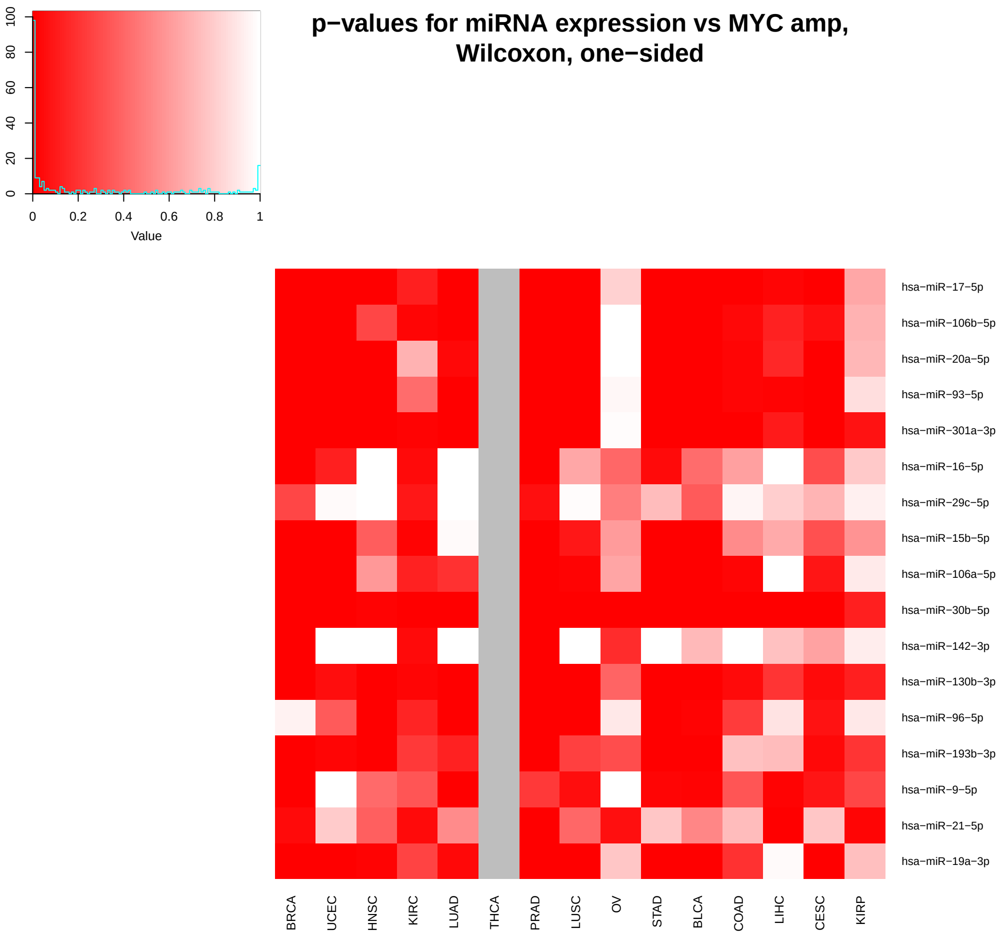

Supplementary Figure 48. Heatmap of the p values obtained for the Wilcoxon rank-sum test, one-sided, comparing TSG-associated miRNA expression for MYC amplified and non-amplified cases, across tumour types (alternative hypothesis miRNA expression greater in amplified cases). NA values are indicated in grey.

Supplementary References

1. A. Dhawan, A. Barberis, W.-C. Cheng, E. Domingo, C. West, T. Maughan, J. Scott, A. L. Harris, and F. M. Buffa. sigQC: A procedural approach for standardising the evaluation of gene signatures. Preprint at https://www.biorxiv.org/content/early/2017/11/13/203729, 2018.
